# Oxytocin stimulates hippocampal neurogenesis via oxytocin receptor expressed in CA3 pyramidal neurons

**DOI:** 10.1038/s41467-017-00675-5

**Published:** 2017-09-14

**Authors:** Yu-Ting Lin, Chien-Chung Chen, Chiung-Chun Huang, Katsuhiko Nishimori, Kuei-Sen Hsu

**Affiliations:** 1 Department of Pharmacology, College of Medicine, National Cheng Kung University, Tainan, 70101 Taiwan; 2 Institute of Basic Medical Sciences, College of Medicine, National Cheng Kung University, Tainan, 70101 Taiwan; 3 Department of Molecular and Cell Biology, Graduate School of Agricultural Science, Tohoku University, Miyagi, 981-8555 Japan; 4 Ph.D. Program for Neural Regenerative Medicine, College of Medical Science and Technology, Taipei Medical University, Taipei, 11031 Taiwan

## Abstract

In addition to the regulation of social and emotional behaviors, the hypothalamic neuropeptide oxytocin has been shown to stimulate neurogenesis in adult dentate gyrus; however, the mechanisms underlying the action of oxytocin are still unclear. Taking advantage of the conditional knockout mouse model, we show here that endogenous oxytocin signaling functions in a non-cell autonomous manner to regulate survival and maturation of newly generated dentate granule cells in adult mouse hippocampus via oxytocin receptors expressed in CA3 pyramidal neurons. Through bidirectional chemogenetic manipulations, we also uncover a significant role for CA3 pyramidal neuron activity in regulating adult neurogenesis in the dentate gyrus. Retrograde neuronal tracing combined with immunocytochemistry revealed that the oxytocin neurons in the paraventricular nucleus project directly to the CA3 region of the hippocampus. Our findings reveal a critical role for oxytocin signaling in adult neurogenesis.

## Abstract (web summary)

Oxytocin (OXT) has been implicated in adult neurogenesis. Here the authors show that CA3 pyramidal cells in the adult mouse hippocampus express OXT receptors and receive inputs from hypothalamic OXT neurons; activation of OXT signaling in CA3 pyramidal cells promotes the survival and maturation of newborn neurons in the dentate gyrus in a non-cell autonomous manner.

## Introduction

Oxytocin (OXT) is a nine amino acid neuropeptide that is primarily synthesized in magnocellular neurons of the hypothalamic paraventricular (PVN) and supraoptic nuclei (SON)^1, 2^. Apart from its release into the systemic circulation via the posterior pituitary, OXT is also transported axonally from hypothalamic parvocellular neurons to numerous extra-hypothalamic OXT receptor (OXTR)-expressing brain regions, including the hippocampus, amygdala, lateral septum, striatum, and bed nucleus of the stria terminals, acting as either a neuromodulator or neurotransmitter to regulate neurotransmission within these regions^3–7^. OXT has both peripheral and central functions. Peripheral OXT promotes uterine contractions during parturition and milk ejection during lactation^8, 9^. Centrally acting OXT has been shown to regulate various social (e.g., aggression, affiliation, bonding, and social recognition) and nonsocial behaviors (e.g., anxiety, stress, depression, and learning and memory)^6, 10–12^. Furthermore, OXT mediates its biological activities by binding to the OXTR, which belongs to the superfamily of G-protein-coupled receptors^2^.

Growing evidence suggests that the hippocampus is one of the brain structures particularly vulnerable to the effects of OXT. For example, microinjection of OXT into the dorsal hippocampus has been shown to attenuate stress-induced neuroendocrine and behavioral responses in rats^13^. In addition, we and others have previously shown that OXT can promote the maintenance of long-term potentiation (LTP) in hippocampal CA1 region and enhance spatial memory during motherhood^14, 15^. The OXT-induced enhancement of LTP is associated with a rapid and persistent increase in dendritic protein kinase Mζ protein synthesis via a mammalian target of rapamycin-mediated mechanism^15^. Moreover, Tsien and his colleagues have recently shown that OXT can enhance hippocampal spike transmission by increasing fast-spiking GABAergic interneuron activity to improve the performance of neural circuitry that demands synaptic specificity and temporal precision^16^. Despite these study points toward a crucial role for OXT in facilitating hippocampal plasticity and function, exogenous application of OXT has been reported to exert a neurotrophic effect to increase adult neurogenesis even when experienced to stressful situations^17^. Nonetheless, how OXT regulates adult neurogenesis remains an unresolved problem. Furthermore, it is not yet clear whether endogenous OXT signaling may also play a role in regulating adult hippocampal neurogenesis. In this study, we addressed the following three questions. First, is OXTR expressed in adult hippocampal neural progenitor cells? Second, does endogenous OXT signaling regulates specific stages of adult hippocampal neurogenesis? Finally, does endogenous OXT signaling regulates adult neurogenesis through a cell autonomous or non-cell autonomous mechanism? Using a Cre/loxP recombinase-based strategy to delete *Oxtr*, we report here that endogenous OXT signaling controls adult hippocampal neurogenesis through an indirect non-cell autonomous mechanism by OXTR expressed in CA3 pyramidal neurons.

## Results

### OXTR is not expressed in the neural progenitor cells

**Fig. 1:**
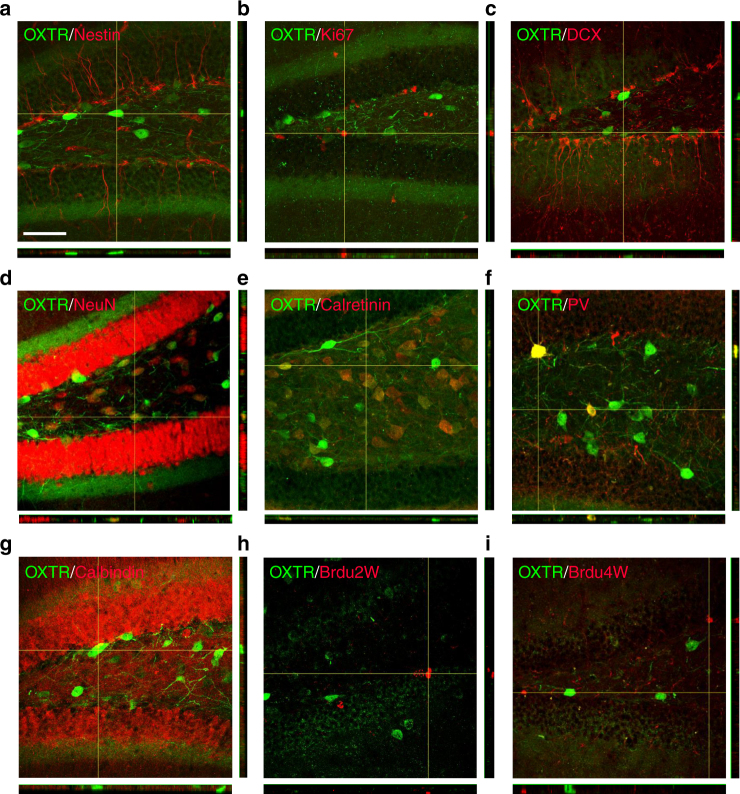
Expression of OXTR in the DG. **a**–**g** Double-labeled confocal immunofluorescence images showing the colocalization of OXTR (*green*) expression with **a** nestin (*red*), **b** Ki67 (*red*), **c** DCX (*red*), **d** NeuN (*red*), **e** calretinin (*red*), **f** parvalbumin (*PV*, *red*), and **g** calbindin (*red*) in the DG of 10-week-old *Oxtr* ^Venus-Neo/+^ mice. **h**, **i** Immunofluorescent staining of BrdU. *Oxtr* ^Venus-Neo/+^ mice were injected with BrdU and killed 2 or 4 weeks later. Representative images of BrdU^+^ cells at **h** 2 week and **i** 4 weeks. Data was replicated in four mice. *Scale bar*, 50 μm

Adult neurogenesis is a multistep process that comprises the proliferation of neural progenitor cells, newborn neuron differentiation, maturation, and functional integration into the preexisting neural networks^18, 19^. We first examined OXTR expression in developing dentate granule cells (DGCs) in adult mouse hippocampus by staining for proteins expressed at distinct stages of cell differentiation. Because an immunofluorescent staining using antibody is not a reliable technique for identifying OXTR-expressing neurons in mice^20^, we therefore used OXTR-Venus knock-in (*Oxtr* ^Venus-Neo/+^) mice to characterize OXTR-expressing neurons in the hippocampus. In 10-week-old mice, unexpectedly, we found few or no Venus-positive cells in the dentate gyrus (DG) expressing progenitor markers, such as nestin (Fig. 1a), Ki67 (Fig. 1b), or neuroblast and immature neuronal marker doublecortin (DCX; Fig. 1c). However, in virtually all Venus-positive cells coexpressed the mature neuronal-specific nuclear protein (NeuN; Fig. 1d). Immunofluorescence staining was also performed with antibodies against numerous known marker proteins expressed in the mature granule cell (calbindin) and the subtypes of GABAergic interneurons (calretinin and parvalbumin). In the hilus, 77.4 ± 2.3% of Venus-positive cells expressed calretinin immunoreactivity (Fig. 1e) and 25.0 ± 2.9% Venus-positive cells expressed parvalbumin immunoreactivity (Fig. 1f). However, no Venus-positive cells were found that expressed calbindin (Fig. 1g). To label proloferating cells, we administered a synthetic analog of the nucleoside thymidine 5-bromo-2′-deoxyuridine (BrdU) intraperitoneally and analyzed BrdU incorporation 2 or 4 weeks later. We detected no Venus-positive cells expressing BrdU immunoreactivity (Fig. 1h, i). We further characterize hilar and CA3 OXTR distribution regarding to overlap with GABA immunoreactivity. In the hilus, 27.8 ± 3.8% of Venus-positive cells were immunoreactive for the GABAergic marker glutamic acid decarboxylase (GAD)67. In the CA3, 3.8 ± 0.7% of Venus-positive cells expressed GAD67 immunoreactivity (Supplementary Fig. 1a, b). These results indicate that OXTR is not expressed in the neural progenitor cells that reside within the subgranular zone or mature granule cells of adult DG and a small subset of hilar GABAergic neurons express OXTR.

### Deletion of *Oxtr* from hippocampal excitatory neurons

**Fig. 2:**
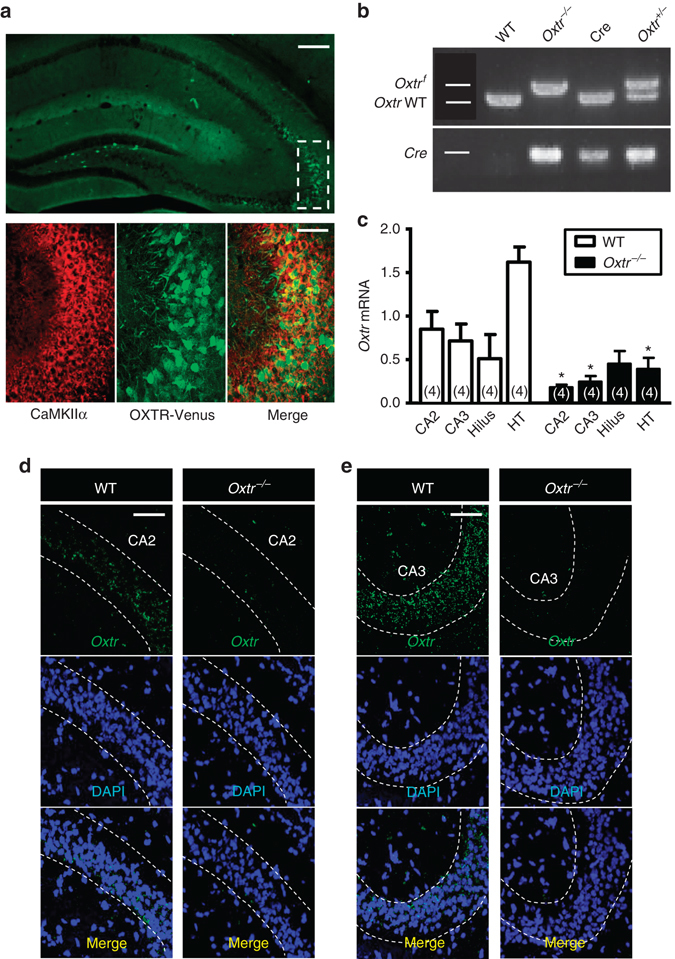
Conditional deletion of *Oxtr* in the excitatory neurons of mouse hippocampus. **a** *Top panel*, a representative immunofluorescence image of OXTR (*green*) expression in the mouse hippocampus. *Bottom panels*, augmented figures showing CaMKIIα (*red*) and OXTR (*green*) double-labeling results in *rectangle area*. *Scale bars*: *top*, 200 μm; *bottom*, 50 μm. **b** PCR screening of tail-derived genomic DNA for selection of *Oxtr* ^*−/−*^ mice. **c** Quantitative real-time PCR of *Oxtr* mRNA in the CA2, CA3, hilus, and hypothalamus (*HT*) regions from WT (*Oxtr* ^*f/f*^) and *Oxtr* ^*−/−*^ mice (*n* = 4 mice for each group; *P < 0.05, unpaired two-tailed Student’s *t* test). **d**, **e** FISH images showing the expression of *Oxtr* mRNA in the CA2 **d** and CA3 regions **e** of WT and *Oxtr* ^*−/−*^ mice (counterstained with DAPI, *blue*). *Scale bar*, 50 μm. Data was replicated in four mice

Previous studies have shown that CA3 pyramidal neurons send reciprocal projections back to the DG and regulate neurogenesis in the DG of adult rats^21^. Since OXTR is enriched in the CA2 and CA3 of the hippocampus^20^, we therefore hypothesized that OXT may control adult hippocampal neurogenesis via OXTR expressed in CA3 pyramidal neurons. Consistent with previous findings^20^, our immunofluorescent staining data showed strong Venus immunoreactivity in the hippocampal CA3 region of *Oxtr* ^Venus-Neo/+^ mice. We found that more than 95% Venus-positive cells expressed calcium/calmodulin-dependent protein kinase II α (CaMKIIα) immunoreactivity (Fig. 2a), indicating that OXTR predominantly expressed in excitatory pyramidal neurons. To test whether OXTR expressed in CA3 pyramidal neurons plays a role in regulating adult DG neurogenesis, we used the Cre-loxP recombination approach to conditionally delete *Oxtr* from hippocampal excitatory neurons by crossing mice expressing CaMKIIα-Cre with mice in which *Oxtr* is floxed (*Oxtr* ^*f/f*^). Polymerase chain reaction (PCR) screening of mouse genomic tail DNA confirmed heterozygous (*Oxtr* ^+/−^) and homozygous *Oxtr* (*Oxtr* ^*−/−*^) conditional knockout mice (Fig. 2b). Quantitative real-time PCR analysis confirmed a reduction in *Oxtr* mRNA expression in the CA2, CA3, and hypothalamus, but not the hilus of the DG, in *Oxtr* ^*−/−*^ mice compared with wild-type (WT, *Oxtr* ^*f/f*^) mice (Fig. 2c). In parallel, fluorescence in situ hybridization (FISH) with *Oxtr* gene probe also revealed that the numbers of *Oxtr* mRNA-positive cells in the CA2 and CA3 of *Oxtr* ^*−/−*^ mice were markedly reduced compared with WT mice (Fig. 2d, e), confirming the efficiency of Cre-loxP-mediated deletion of *Oxtr*. Dual-probe FISH also confirmed that the majority of *Oxtr* mRNA-positive cells were *CaMKIIα* mRNA-expressing cells in the CA3 (Supplementary Fig. 2a). Very few *Oxtr* mRNA immunoreactivity was detected in the granule cell layer of the dorsal and ventral DG (Supplementary Fig. 2b, c). Although *Oxtr* mRNA immunoreactivity was detected in dorsal and ventral hilus of the DG, we did not observe colocalization of *Oxtr* mRNA with *CaMKIIα* mRNA. We also demonstrated that *Oxtr* ^*−/−*^ mice specifically displayed a reduction of *Oxtr* mRNA expression in the CA3, but not the hilus of the DG, compared with WT mice (Supplementary Fig. 2).

### Effects of *Oxtr* deletion on newly generated DGCs

**Fig. 3:**
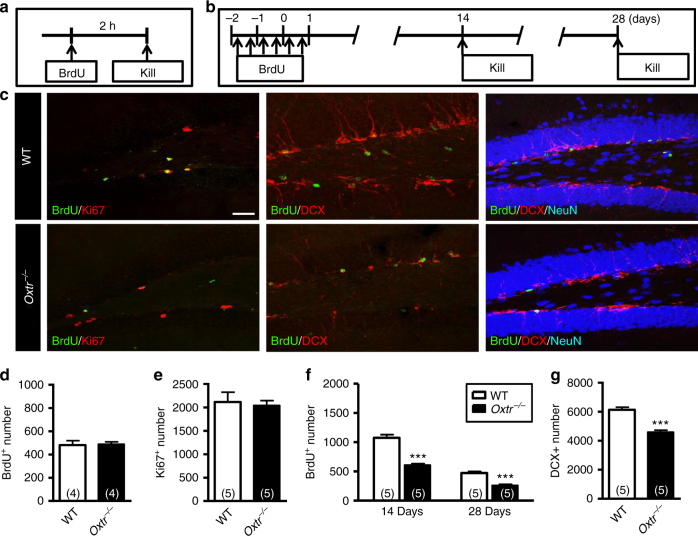
Conditional deletion of *Oxtr* impairs the survival of newly generated DGCs. **a** Schematic representation of the experimental designs for comparing the proliferation of newly generated DGCs in WT and *Oxtr* ^*−/−*^ mice. Mice were given a single injection of BrdU (50 mg/kg) and were killed 2 h after BrdU injection. **b** Schematic representation of the experimental designs for comparing the survival of newly generated DGCs in WT and *Oxtr* ^*−/−*^ mice. Mice were injected six times intraperitoneally with BrdU (50 mg/kg) at 12 h intervals and were killed 14 and 28 days after the last BrdU injection. **c** Representative immunofluorescence images of hippocampal sections from WT and *Oxtr* ^*−/−*^ mice double or triple stained for BrdU (*green*), Ki67 (*red*), DCX (*red*), and NeuN (*blue*) after BrdU injection. *Scale bar*, 100 µm. **d** Quantification of the total number of BrdU^+^ cells in the DG of WT and *Oxtr* ^*−/−*^ mice at 2 h after BrdU injection (*n* = 4 mice per genotype, unpaired two-tailed Student’s *t* test). **e** Quantification of the total number of Ki67^+^ cells in the DG of WT and *Oxtr* ^*−/−*^ mice at 2 h after BrdU injection (*n* = 5 mice per genotype, unpaired two-tailed Student’s *t* test). **f** Quantification of the total number of BrdU^+^ cells in the DG of WT and *Oxtr* ^*−/−*^ mice at 14 and 28 days after BrdU injection (*n* = 5 mice for each group; ***P < 0.001, unpaired two-tailed Student’s *t* test). **g** Quantification of the total number of DCX^+^ cells in the DG of WT and *Oxtr* ^*−/−*^ mice at 14 days after the last BrdU injection (*n* = 5 mice per genotype; ***P < 0.001, unpaired two-tailed Student’s *t* test). Data represent the mean ± s.e.m

To determine whether *Oxtr* deletion from hippocampal excitatory neurons may affect the proliferation of newly generated DGCs, WT and *Oxtr* ^*−/−*^ mice were given a single injection of BrdU, and double fluorescent labeling for detection of BrdU-positive (BrdU^+^) proliferating cells (Ki67^+^) was performed on both dorsal and ventral hippocampal sections 2 h later (Fig. 3a, c). Stereological analysis revealed no differences in the total number of either BrdU^+^ (Fig. 3d) or Ki67^+^ cells (Fig. 3e) between WT and *Oxtr* ^*−/−*^ mice at 2 h after the BrdU injection in both dorsal and ventral hippocampus (Supplementary Fig. 3a–d). The percentage of BrdU^+^Ki67^+^ cells to total BrdU^+^ cells was not different between the two groups (Supplementary Fig. 3i). To determine whether conditional deletion of *Oxtr* from hippocampal excitatory neurons may affect the survival of newly generated DGCs, WT and *Oxtr* ^*−/−*^ mice were subjected to multiple BrdU injections, and double fluorescent labeling for detection of BrdU^+^ immature (DCX^+^) or mature neuronal cells (NeuN^+^) was performed on hippocampal sections 14 or 28 days later (Fig. 3b, c). Interestingly, we found a significant reduction in the total number of BrdU^+^ cells in *Oxtr* ^*−/−*^ mice at 14 and 28 days after BrdU injection compared with WT mice (Fig. 3f). Likewise, a significant reduction in the total number of DCX^+^ cells in *Oxtr* ^*−/−*^ mice at 14 days after BrdU injection compared with WT mice in both dorsal and ventral hippocampus (Fig. 3g and Supplementary Fig. 3e–h). However, the percentage of BrdU^+^DCX^+^ cells or BrdU^+^NeuN^+^ cells to total BrdU^+^ cells was not different between two groups (Supplementary Fig. 3j, k). These results suggest that OXTR plays a non-cell autonomous role in controlling the survival of newly generated DGCs, whereas the progenitor cell proliferation and early differentiation into immature neurons were unaltered by *Oxtr* deletion.

**Fig. 4:**
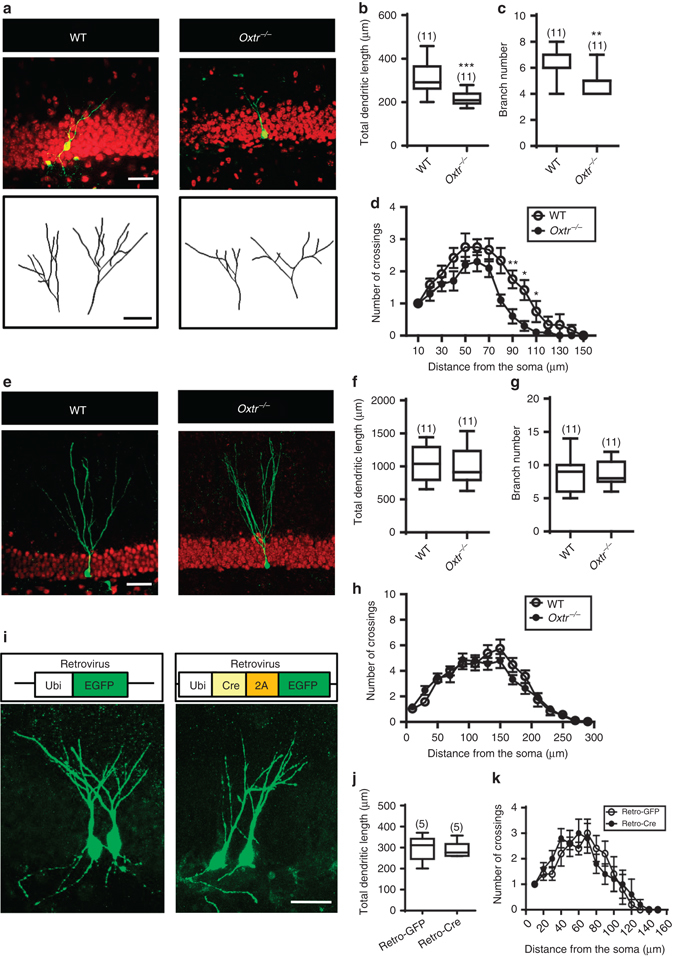
Conditional deletion of *Oxtr* delays the morphological maturation of newly generated DGCs. **a** Confocal three-dimensional reconstruction of dendrites of EGFP^+^ DGCs from WT and *Oxtr* ^*−/−*^ mice at 14 dpi. *Scale bar*, 50 µm. **b**, **c** Summary bar graphs depicting **b** the total dendritic length and **c** branch number of EGFP^+^ DGCs from WT and *Oxtr* ^*−/−*^ mice at 14 dpi (*n* = 11 neurons from 4 mice per genotype; **P < 0.01, ***P < 0.001, unpaired two-tailed Student’s *t* test). **d** Sholl analysis of dendritic complexity of EGFP^+^ DGCs from WT and *Oxtr* ^*−/−*^ mice at 14 dpi (*n* = 11 neurons from 4 mice per genotype; *p < 0.05, **P < 0.01, two-way ANOVA with Bonferroni’s *post hoc* test). **e** Representative confocal images showing EGFP^+^ DGCs from WT and *Oxtr* ^*−/−*^ mice at 28 dpi. *Scale bar*, 50 µm. **f**, **g** Summary bar graphs depicting **f** the total dendritic length and **g** branch number of EGFP^+^ DGCs from WT and *Oxtr* ^*−/−*^ mice at 28 dpi (*n* = 11 neurons from 5 mice per genotype; unpaired two-tailed Student’s *t* test). **h** Sholl analysis of dendritic complexity of EGFP^+^ DGCs from WT and *Oxtr* ^*−/−*^ mice at 28 dpi (*n* = 11 neurons from 5 mice per genotype; two-way ANOVA). **i** *Top panel*, schematic representations of the retroviral vectors used for in vivo genetic manipulation under the control of the Ubi-1 promoter. *Bottom panels*, representative confocal images showing Retro-EGFP^+^ and Retro-Cre-2A-EGFP^+^ DGCs in *Oxtr* ^*f/f*^ mice at 14 dpi. *Scale bar*, 50 µm. **j** Summary bar graphs depicting the total dendritic length of Retro-EGFP^+^ and Retro-Cre-2A-EGFP^+^ DGCs in *Oxtr* ^*f/f*^ mice at 14 dpi (*n* = 5 neurons from 3 mice for each group, unpaired two-tailed Student’s *t* test). **k** Sholl analysis of dendritic complexity of Retro-EGFP^+^ and Retro-Cre-2A-EGFP^+^ DGCs in *Oxtr* ^*f/f*^ mice at 14 dpi (*n* = 5 neurons from 3 mice for each group, two-way ANOVA). Data represent the mean ± s.e.m

We next evaluated the impact of *Oxtr* deletion on the morphological maturation of newly generated DGCs by using a retrovirus-mediated birth-dating and labeling strategy^22^. Engineered retroviruses expressing enhanced green fluorescent protein (EGFP) were stereotaxically microinjected into the DG hilus of WT and *Oxtr* ^*−/−*^ mice. Sholl analysis for dendritic complexity of EGFP-positive (EGFP^+^) DGCs was carried out 14 or 28 days post infection (dpi). Using confocal microscopy to reconstruct the dendritic arborization of EGFP^+^ DGCs, we found that newly generated DGCs from *Oxtr* ^*−/−*^ mice exhibited less elaborated dendritic arborization than those from WT mice at 14 dpi (Fig. 4a). Unpaired Student’s *t* test revealed a significant reduction in both total dendritic length (*P* < 0.001; Fig. 4b) and branch number (*P* < 0.01; Fig. 4c) in EGFP^+^ DGCs from *Oxtr* ^*−/−*^ mice compared to those from WT mice. Sholl analysis further revealed a significant decrease in the dendritic complexity of EGFP^+^ DGCs in *Oxtr* ^*−/−*^ mice compared to WT mice at 14 dpi, an effect more pronounced for the dendritic branches extended beyond 90–110 μm from the soma (Fig. 4d). However, we observed no significant differences in total dendritic length, branch number, or dendritic complexity of EGFP^+^ DGCs between *Oxtr* ^*−/−*^ and WT mice at 28 dpi (Fig. 4e–h).

To further confirm OXT controls adult hippocampal neurogenesis through an indirect non-cell autonomous mechanism, we therefore examined morphological maturation of newly generated DGCs by using *Oxtr* ^*f/f*^ mice in combination with Cre recombinase-mediated gene deletion in a localized fashion through bilateral stereotaxic injections of Cre-expressing (Retro-Cre-EGFP) or control EGFP-expressing (Retro-EGFP) retroviral vectors targeting the hilus of the DG (Fig. 4i). We observed no significant differences in total dendritic length or dendritic complexity of EGFP^+^ DGCs between two groups at 14 dpi (Fig. 4j, k).

**Fig. 5:**
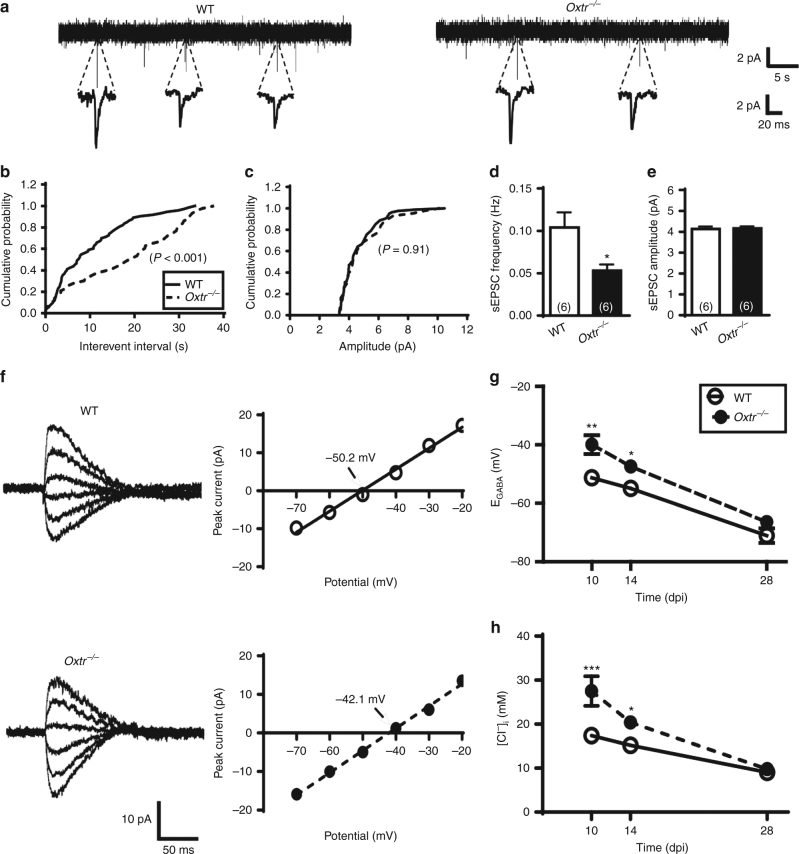
Effects of *Oxtr* deletion on excitatory and inhibitory synaptic transmission of newly generated DGCs. **a** Representative traces of sEPSCs recorded from newly generated DGCs in slices from WT and *Oxtr* ^*−/−*^ mice at 14 dpi. **b**, **c** Cumulative probability plots of sEPSC **b** interevent intervals and **c** amplitude (*n* = 6 neurons from 4 mice per genotype, Kolmogorov-Smirnov test) in newly generated DGCs from WT and *Oxtr* ^*−/−*^ mice at 14 dpi. **d**, **e** Summary bar graphs depicting the averaged **d** frequency and **e** amplitude of sEPSCs in newly generated DGCs from WT and *Oxtr* ^*−/−*^ mice at 14 dpi (*n* = 6 neurons from 4 mice per genotype; *P < 0.05, unpaired two-tailed Student’s *t* test). **f** *Left panel*, Sample traces of evoked IPSCs recorded at different holding potentials ranging from −70 to −20 mV (10 mV step) from newly generated DGCs in slices from WT and *Oxtr* ^*−/−*^ mice at 10 dpi. *Right panel*, current-voltage plot of the peak current of evoked IPSCs obtained from the sample traces. **g** Summary data comparing the reversal potential of GABA-mediated IPSCs (E_GABA_) of newly generated DGCs from WT and *Oxtr* ^*−/−*^ mice at 10, 14, and 28 dpi (*n* = 4–5 neurons from 4 mice for each group; *P < 0.05, **P < 0.01, two-way ANOVA with Bonferroni’s post hoc test). **h** Summary data comparing the calculated intracellular chloride concentrations ([Cl^−^]_i_) in newly generated DGCs from WT and *Oxtr* ^*−/−*^ mice at 10, 14, and 28 dpi (*n* = 4–5 neurons from 4 mice for each group; *P < 0.05, ***P < 0.001, two-way ANOVA with Bonferroni’s post hoc test). Data represent the mean ± s.e.m

To assess whether the morphological changes that resulted from conditional *Oxtr* deletion may lead to alterations in physiological properties, we performed electrophysiological analysis of newly generated DGCs in acute slices from retrovirus-injected mice at 14 dpi. We first examined glutamatergic synaptic transmission by recording spontaneous excitatory postsynaptic currents (sEPSCs) in EGFP^+^ DGCs in the presence of GABA_A_ receptor antagonist gabazine (10 μM; Fig. 5a). A significant difference in cumulative interevent interval distribution was observed between newly generated DGCs from *Oxtr* ^*−/−*^ and WT mice (Fig. 5b). However, no significant difference in cumulative amplitude distribution was observed between two groups (Fig. 5c). The mean frequency of sEPSCs in EGFP^+^ DGCs from *Oxtr* ^*−/−*^ mice was significantly less than those from WT mice (*P* < 0.05; Fig. 5d). No significant difference was observed between *Oxtr* ^*−/−*^ and WT mice in the mean amplitude of sEPSCs (*P* = 0.88; Fig. 5e).

The newly generated DGCs exhibit initial depolarizing response to GABA, which gradually shift to hyperpolarizing responses over a period of 2–3 weeks of birth^23^. The polarity of GABA actions depends on the chloride concentration gradient across the plasma membrane. We next used gramicidin-perforated patch-clamp recordings to determine the nature of GABA activation by recording evoked inhibitory postsynaptic currents (IPSCs) in EGFP^+^ DGCs from *Oxtr* ^*−/−*^ and WT mice at 10, 14, and 28 dpi (Fig. 5f). In agreement with previous findings^23^, we found that the reversal potential for GABA_A_ receptor-mediated IPSCs (E_GABA_) in EGFP^+^ DGCs gradually decreased during maturation. Two-way ANOVA revealed a main effect of conditional *Oxtr* deletion on E_GABA_ (*F*_(1,18)_ = 25.1, *P* < 0.001; Fig. 5g). Bonferroni’s post hoc tests showed that EGFP^+^ DGCs from *Oxtr* ^*−/−*^ mice demonstrated more positive values for E_GABA_ compared to those from WT mice at both 10 (*P* < 0.01) and 14 dpi (*P* < 0.05). To determine whether more positive values for E_GABA_ could be due to higher intracellular chloride concentrations ([Cl^−^]_i_), we used the Nernst equation to calculate [Cl^−^]_i_. Two-way ANOVA revealed a main effect of conditional *Oxtr* deletion on [Cl^−^]_i_ (*F*_(1,18)_ = 23.7, *P* < 0.01; Fig. 5h). Bonferroni’s post hoc tests showed that GFP^+^ DGCs from *Oxtr* ^*−/−*^ mice had higher [Cl^−^]_i_ than those from WT mice at both 10 (*P* < 0.0001) and 14 dpi (*P* < 0.01). These results indicate that conditional *Oxtr* deletion may delay maturation of newly generated DGCs.

**Fig. 6:**
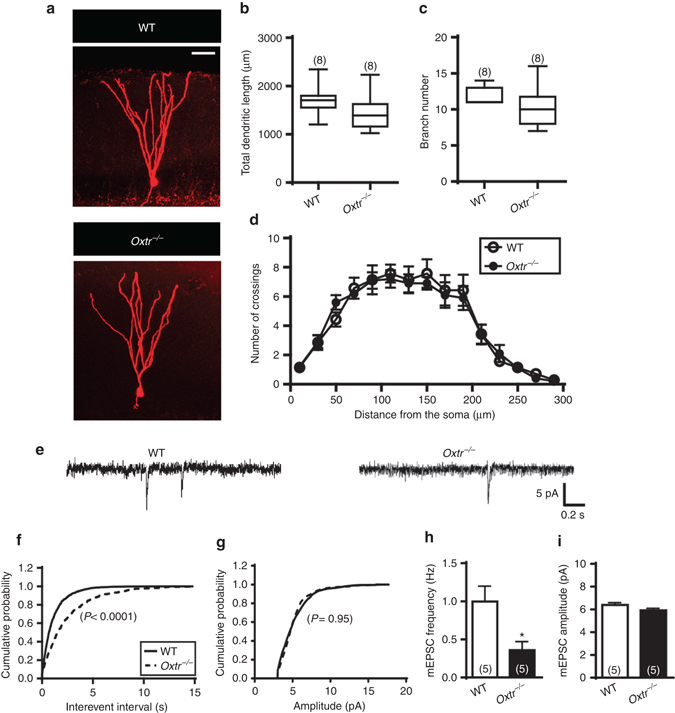
Effects of *Oxtr* deletion on morphological features and excitatory synaptic transmission of mature DGCs. **a** Representative micrographs of biocytin-filled mature DGCs from WT and *Oxtr* ^*−/−*^ mice. *Scale bar*, 50 μm. **b**, **c** Summary bar graphs depicting **b** the total dendritic length and **c** branch number of mature DGCs from WT and *Oxtr* ^*−/−*^ mice (*n* = 8 neurons from 5 mice per genotype; unpaired two-tailed Student’s *t* test). **d** Sholl analysis of dendritic complexity of mature DGCs from WT and *Oxtr* ^*−/−*^ mice (*n* = 8 neurons from 5 mice per genotype, two-way ANOVA). **e** Representative traces of mEPSCs recorded from mature DGCs in slices from WT and *Oxtr* ^*−/−*^ mice. **f**, **g** Cumulative probability plots of mEPSC **f** interevent intervals and **g** amplitude (*n* = 5 neurons from 3 mice per genotype, Kolmogorov-Smirnov test) in mature DGCs from WT and *Oxtr* ^*−/−*^ mice. **h**, **i** Summary bar graphs depicting the averaged **h** frequency and **i** amplitude of mEPSCs in mature DGCs from WT and *Oxtr* ^*−/−*^ mice (*n* = 5 neurons from 3 mice per genotype; *P < 0.05, unpaired two-tailed Student’s *t* test). Data represent the mean ± s.e.m

We next evaluated whether *Oxtr* deletion impacted mature DGCs. To characterize the morphological and electrophysiological properties of DGCs, we recorded miniature excitatory postsynaptic currents (mEPSCs) from mature DGCs with the use of whole-cell patch-clamp recordings and biocytin was routinely included in the intracellular solution to allow post hoc identification of the recorded neurons (Fig. 6a). We observed no significant differences between *Oxtr* ^*−/−*^ and WT mice in total dendritic length (*P* = 0.19; Fig. 6b) and branch number of DGCs (*P* = 0.22; Fig. 6c). Sholl analysis revealed no significant difference in the dendritic complexity of DGCs between *Oxtr* ^*−/−*^ and WT mice (*F*_(1,225)_ = 0.14, *P* = 0.71; Fig. 6d). mEPSCs were recorded in the presence of tetrodotoxin (TTX, 0.5 μM) and gabazine (10 μM; Fig. 6e). A significant difference in cumulative interevent interval distribution was observed between mature DGCs from *Oxtr* ^*−/−*^ and WT mice (Fig. 6f). However, no significant difference in cumulative amplitude distribution was observed between two groups (Fig. 6g). The mean frequency of mEPSCs in DGCs from *Oxtr* ^*−/−*^ mice was significantly less than those from WT mice (*P* < 0.05; Fig. 6h). No significant difference was observed between *Oxtr* ^*−/−*^ and WT mice in the mean amplitude of mEPSCs (*P* = 0.11; Fig. 6i).

### OXT controls adult neurogenesis through CA3 neurons

**Fig. 7:**
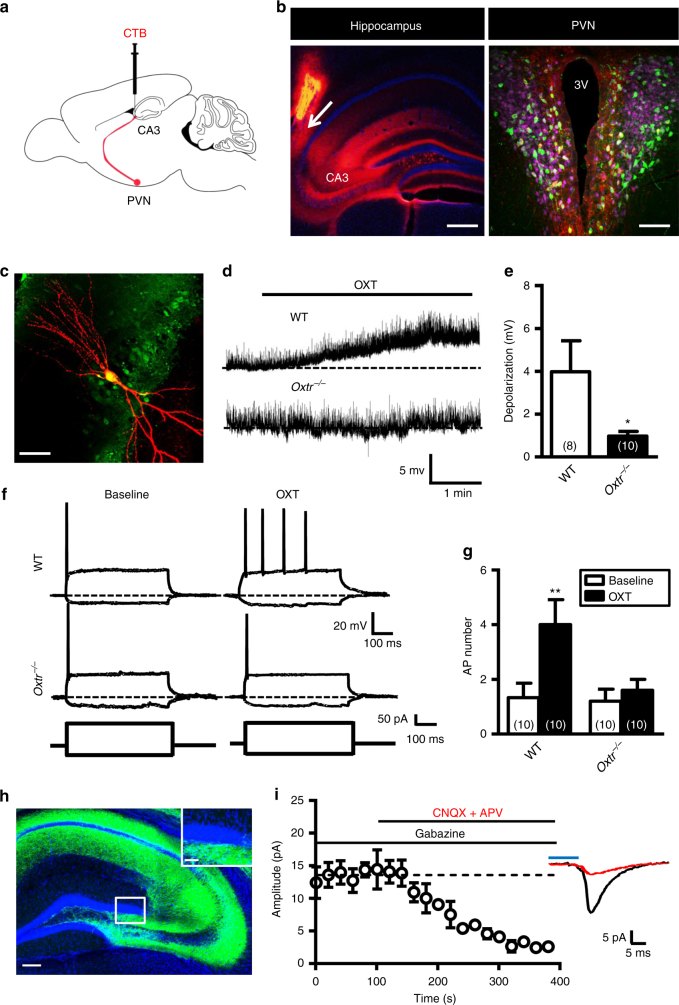
OXT increases activity of CA3 pyramidal neurons. **a** A schematic of the approach to label PVN-projecting OXT neurons by injection of CTB-555 into the hippocampal CA3 region and intraperitoneal injection of fluoro-gold (4% w/v in 100 μl saline). **b** *Left panel*, CA3 pyramidal neurons labeled by CTB-555 (*red*). Section was counterstained with DAPI (*blue*). *Right panel*, PVN neurons back-labeled from the hippocampal CA3 region. The CTB^+^ cell bodies (*red*) were found in the caudal portion of the PVN and colocalized OXT (*green*) and fluoro-gold (*pink*). The *arrow* indicates the site of injection. Data were replicated in four mice. *Scale bars*: *left*, 250 μm; *right*, 100 μm. **c** OXTR-containing CA3 pyramidal neuron was targeted for recording, filled with biocytin, and reacted with avidin-rhodamine. *Scale bar*: 20 μm. **d**, **e** Representative traces and summary data comparing OXT (1 μM)-induced membrane depolarization in hippocampal CA3 pyramidal neurons from WT and *Oxtr* ^*−/−*^ mice (WT, *n* = 8 neurons from 6 mice; *Oxtr* ^*−/−*^, *n* = 10 neurons from 6 mice; *P < 0.05, unpaired two-tailed Student’s *t* test). **f**, **g** Representative traces and summary data comparing the effect of OXT (1 μM) on action potential firing responses elicited by depolarizing current injection in hippocampal CA3 pyramidal neurons from WT and *Oxtr* ^*−/−*^ mice (*n* = 10 neurons from 6 mice per genotype; **P < 0.01, paired two-tailed Student’s *t* test). **h** Representative micrographs showing the expression of ChR2-EYFP in the subgranular zone of the DG and stratum radiatum. *Scale bars*: 100 and 20 μm (rectangle amplification). **i** Summary graph showing the average amplitude of optically evoked EPSCs recorded in DGCs after application of gabazine (10 μM) and CNQX (20 μM) plus APV (50 μM) (*n* = 3 neurons). Data represent the mean ± s.e.m

Having established that conditional deletion of *Oxtr* results in impaired survival and maturation of newly generated DGCs, we next investigated the possible mechanisms by which OXT controls adult hippocampal neurogenesis. Based on the foregoing observations in conjunction with the suggested role of CA3 pyramidal neurons in regulating adult neurogenesis, we tested whether OXT neurons of the PVN send projections to the CA3 region of the hippocampus. To identify PVN-CA3 projection neurons, we injected a monosynaptic retrograde tracer cholera toxin B subunit (CTB) into the CA3 and examined the distribution of retrogradely CTB-labeled cells appeared in the PVN (Fig. 7a, b). We also labeled neurosecretory neurons with intraperitoneal injection of the retrograde tracer, fluoro-gold (4% w/v in 100 μl saline)^24^. Immunoreactivity for CTB was clearly detected in fluoro-gold^−^ and OXT^+^ parvocellular neurons of the PVN (Fig. 7b), but not the SON (Supplementary Fig. 4a). We also observed that PVN neurons send their projecting fibers directly to the CA3 (Supplementary Fig. 4b). These results indicate that the CA3 contains a dense population of OXTR-expressing neurons and receives direct inputs from OXT neurons in the PVN.

To demonstrate that CA3 pyramidal neurons are functionally modulated by OXT, we analyzed the effects of OXT on electrical membrane properties of CA3 neurons (Fig. 7c). Under whole-cell current-clamp condition, bath application of OXT (1 μM) consistently caused a membrane depolarization by 3.98 ± 1.44 mV (*n* = 8) in CA3 pyramidal neurons from WT mice. The OXT-induced membrane depolarization was not observed in CA3 pyramidal neurons from *Oxtr* ^*−/−*^ mice (0.98 ± 0.21 mV, *n* = 10; Fig. 7d, e) and was prevented by the selective OXTR antagonist L-371257 (1 μM, Supplementary Fig. 5a, b), indicating that such action is mediated by the OXTR. Furthermore, consistent with increased CA3 pyramidal neuron excitability, we found that the firing activity of CA3 neurons by quantifying the number of action potentials in response to postsynaptic depolarizing current injection (100 pA, 500 ms) was increased after OXT application (4.0 ± 0.9 spikes, *n* = 10, *P* < 0.05) compared with vehicle-treated controls (1.3 ± 0.8 spikes, *n* = 10) in WT mice (Fig. 7f, g). However, bath application of OXT failed to elicit any significant change in the number of action potentials in response to postsynaptic depolarizing current injection in CA3 pyramidal neurons from *Oxtr* ^*−/−*^ mice (1.6 ± 1.3 spikes, *n* = 10, *P* = 0.22) compared with vehicle-treated controls (1.2 ± 0.8 spikes, *n* = 10). The enhancing effect of OXT on action potential firing in CA3 pyramidal neurons was also prevented by the pretreatment of the hippocampal slices with L-371257 (1 μM; Supplementary Fig. 5c, d). To further validate whether OXT may act indirectly to enhance intrinsic excitability of hippocampal interneurons through activation of CA3 OXTR^+^ pyramidal neurons, CA3 OXTR^−^ interneurons in slices from *Oxtr* ^Venus-Neo/+^ mice were targeted for recording, filled with biocytin to allow post hoc reconstruction (Supplementary Fig. 6a). In contrast to what has been observed in CA3 OXTR^+^ pyramidal neurons, bath application of OXT (1 μM) did not significantly affect membrane potential in the absence of presence of L-371257 (1 μM) in CA3 OXTR^−^ interneurons (Supplementary Fig. 6b–d).

We employed optogenetic approach to further validate that DGCs receive a direct back projection from CA3 pyramidal neurons. We unilaterally injected the CA3 region with a recombinant adeno-associated virus capsid DJ (AAV-DJ) serotype expressing the light-sensitive channelrhodopsin 2 (ChR2) tagged with enhanced yellow fluorescent protein (EYFP), under control of the CaMKIIα promoter [AAV-CaMKIIα-hChR2(H134R)-EYFP], favoring expression within excitatory neurons^25^. Acute brain slices were prepared 3 weeks after virus injection. Our immunohistochemical analysis revealed robust and unilateral expression of EYFP in the subgranular zone of the DG and the stratum radiatum (Fig. 7h). We used whole-cell recording to examine postsynaptic currents in DGCs while optically stimulating ChR2-EYFP-positive projections in the DG. Blue light pulses reliably induced postsynaptic currents in DGCs. These optically evoked postsynaptic currents were not significantly affected by gabazine (10 μM) but were blocked by concomitant application of 6-cyano-7-nitroquinoxaline-2, 3-dione (CNQX; 20 μM) and D-2-amino-5-phosphonopentanoic acid (APV; 50 μM), indicating that they were excitatory postsynaptic responses (Fig. 7i). We confirmed that DGCs indeed receive a direct back projection from CA3 pyramidal neurons.

**Fig. 8:**
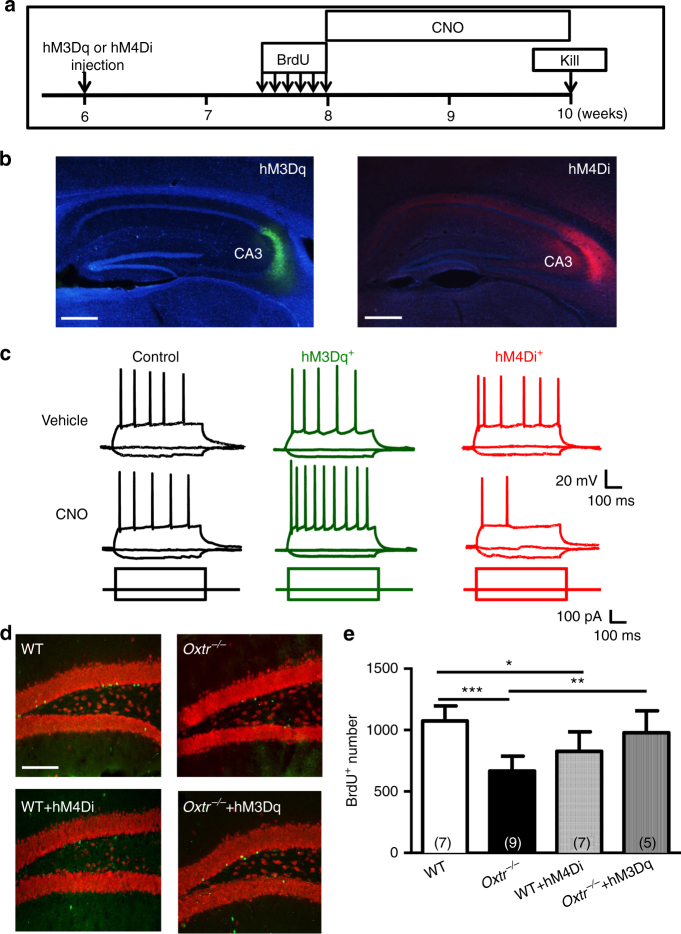
CA3 pyramidal neuronal activity regulates adult hippocampal neurogenesis. **a** Schematic representation of the experimental design. One week after stereotaxic injection of AAV-hM3Dq or AAV-hM4Di into the CA3 region, mice were injected six times intraperitoneally with BrdU (50 mg/kg) at 12 h intervals. After daily intraperitoneal injection of CNO (10 mg/kg) for 2 weeks, mice were killed and BrdU incorporation was measured. **b** Representative micrographs showing the expression of (*left panel*) hM3Dq and (*right panel*) hM4Di receptors in the hippocampal CA3 region (counterstained with DAPI, *blue*). *Scale bar*: 500 μm. **c** Representative traces showing responses of uninfected (control) and infected (hM3Dq^+^ or hM4Di^+^) neurons to depolarizing current ( + 150 pA, 500 ms) or hyperpolarizing current pulse (−50 pA, 500 ms) under whole-cell current-clamp before and after bath application of CNO (1 μM) in the ex vivo hippocampal slices. **d** Representative immunofluorescence images of hippocampal sections from WT, *Oxtr* ^*−/−*^, WT + hM4Di and *Oxtr* ^*−/*−^ + hM3Dq stained for BrdU (*green*) at 14 days after the last BrdU injection (counterstained with NeuN, *red*). *Scale bar*, 100 µm. **e** Quantification of the total number of BrdU^+^ cells in the dentate gyrus of WT, *Oxtr* ^*−/−*^, WT + hM4Di and *Oxtr* ^*−/−*^ + hM3Dq mice at 14 days after BrdU injection (WT, *n* = 7 mice; *Oxtr* ^*−/−*^ *, n* = 9 mice; WT + hM4Di, *n* = 7 mice; *Oxtr* ^*−/−*^ *+* hM3Dq, *n* = 5 mice; *P < 0.05, **P < 0.01, and ***P < 0.001, unpaired two-tailed Student’s *t* test). Data represent the mean ± s.e.m

Given that chemogenetics permits bidirectional manipulation of neuronal activity with anatomical, genetic, and temporal precision, we also used in vivo chemogenetic approaches to study the role of CA3 pyramidal neuronal activity in controlling adult hippocampal neurogenesis. The experimental procedure is depicted in Fig. 8a. We bilaterally injected the CA3 region with an AAV-DJ viral vector expressing an engineered Gq-coupled human M3 (hM3Dq) tagged with mCitrine (hM3Dq-mCitrine) or G_i/o_-coupled human M4 (hM4Di) tagged with mCherry (hM4Di-mCherry), under control of the CaMKIIα promoter. One week later, WT and *Oxtr* ^*−/−*^ mice were subjected to multiple BrdU injections and analyzed BrdU incorporation 2 weeks later. Mice were injected intraperitoneally daily with clozapine-*N*-oxide (CNO, 10 mg/kg), a synthetic ligand of hM3Dq and hM4Di^26^, for continuous 2 weeks until killig. Post hoc histological examination of brain sections revealed robust and bilateral expression of hM3Dq or hM4Di in CA3 pyramidal neurons (Fig. 8b). In a subset of mice, we performed ex vivo electrophysiological recordings to confirm the effects of CNO in AAV-infected neurons. Application of CNO (1 μM) significantly decreased spiking responses to +150 pA square current pulses in hM4Di-expressing (hM4Di^+^) neurons but increased spiking responses in hM3Dq-expressing (hM3Dq^+^) neurons (Fig. 8c). In *Oxtr* ^*−/−*^ mice, we found that the total number of BrdU^+^ cells was significantly increased by hM3Dq/CNO-based approach (Fig. 8d, e). Conversely, hM4Di/CNO-based approach led to a decrease in the total number of BrdU^+^ cells in WT mice (Fig. 8d, e). To evaluate whether chronic CNO administration may cause cell death of CA3 pyramidal neurons, we used terminal deoxynucleotidyl transferase-mediated dUTP nick-end labeling (TUNEL) assay to assess the extent of apoptotic death. No TUNEL-positive cells were found in the CA3 of WT mice with or without hM3Dq/CNO treatment compared with DNase-treated positive control (Supplementary Fig. 7), confirming that our chronic CNO treatment protocol does not induce apoptotic cell death.

Since *Oxtr* deletion in *Oxtr* ^*−/−*^ mice was not restricted to the CA3, the loss of OXTR function in other hippocampal or forebrain regions may also contribute to impair the survival and maturation of newly generated DGCs. To circumvent this limitation, in a subset of experiments, we used *Oxtr* ^*f/f*^ mice in combination with Cre recombinase-mediated gene deletion in a localized fashion through bilateral stereotaxic injections of AAV-Cre-GFP or control AAV-GFP vectors targeting the CA3, under control of the CaMKIIα promoter, favoring expression to excitatory neurons. One day before stereotaxic injection of AAV-GFP or AAV-Cre-GFP, mice were subjected to multiple BrdU injections, and the number of BrdU^+^ cells was measured 2 weeks after the last BrdU injection (Supplementary Fig. 8a). The successful transduction of AAV-GFP or AAV-Cre-GFP was confirmed by immunohistochemistry (Supplementary Fig. 8b). Quantitative real-time PCR analysis confirmed the loss of *Oxtr* mRNA expression in the CA3 2 weeks following the stereotactic injection of AAV-Cre-GFP (Supplementary Fig. 8c). Consistent with the results from *Oxtr* ^*−/−*^ mice, we found a significant reduction of the total of BrdU^+^ cells in *Oxtr* ^*f/f*^ mice with AAV-Cre-GFP injections compared with those injected with of AAV-GFP (Supplementary Fig. 8d, e). We also used retroviral approach to label newly generated DGCs in *Oxtr* ^*f/f*^ mice with AAV-GFP or AAV-Cre-GFP injections. Engineered retroviruses expressing EGFP were stereotaxically microinjected into the hilus of the DG 1 day before AAV-GFP or AAV-Cre-GFP treatment, and the dendritic arborization of EGFP^+^ DGCs was measured at 14 dpi (Supplementary Fig. 8f). We confirmed a significant reduction in both dendritic length (Supplementary Fig. 8g, h) and branch number (Supplementary Fig. 8g, i) in EGFP^+^ DGCs from *Oxtr* ^*f/f*^ mice with AAV-Cre-GFP injections compared with those injected with AAV-GFP. Taken together, these results suggest that CA3 neuronal activity plays an important role in regulating adult hippocampal neurogenesis under basal conditions and OXT controls neurogenesis via OXTR expressed in CA3 pyramidal neurons.

## Discussion

In the present study, we examined the role of endogenous OXT signaling in adult hippocampal neurogenesis. There are three major findings. First, OXTR is not expressed in neural progenitor cells reside within the subgranular zone or mature granule cells of adult DG, whereas it is enriched in the CA2 and CA3 of the hippocampus. Second, conditional deletion of *Oxtr* from hippocampal excitatory neurons leads to impaired survival and maturation of newly generated DGCs. Third, OXT enhances the excitability of hippocampal CA3 pyramidal neurons and CA3 neuronal activity regulates adult hippocampal neurogenesis under basal conditions. Together, these results highlight a non-cell autonomous role for OXT in promoting adult hippocampal neurogenesis via OXTR expressed in CA3 pyramidal neurons.

OXTR is widely expressed in the brain. Early studies using radiolabeled ligands or transgenic approaches have demonstrated abundant expression of OXTR in the hippocampus^20, 27^; however, no studies have yet reported whether OXTR is expressed in developing DGCs in adult mouse brain. Using *Oxtr* ^Venus-Neo/+^ mice in conjunction with immunofluorescent staining for proteins expressed at distinct stages of cell differentiation, we demonstrate here for the first time that OXTR is not expressed in neural progenitor cells or mature granule cells of adult mouse DG. Instead, we confirmed in the previous findings that OXTR is highly expressed in hippocampal CA2 and CA3 pyramidal neurons^20^. We also found that OXTR is present in a subset of calretinin-expressing cells in the hilus of the DG. Given that hilar calretinin-expressing cells in mouse DG could belong to either GABAergic interneurons or mossy cells^28^, additional studies are needed to clarify the neuronal identity of hilar OXTR-expressing cells.

A recent study using pharmacological approaches has proposed that OXT could protect the hippocampus from the detrimental effects of elevated glucocorticoids by promoting adult neurogenesis^17^. It has been demonstrated that both acute peripheral and intra-hippocampal OXT administration enhances cell proliferation, and repeated OXT administration increases the number of newly generated DGCs in adult rat hippocampus^17^. We have extended these findings by underscoring the importance of OXTR signaling in the regulation of adult hippocampal neurogenesis under basal conditions. This view is supported by the observation that conditional deletion of *Oxtr* from hippocampal excitatory neurons resulted in impaired survival and maturation of newly generated DGCs. Our data suggest, however, that the progenitor cell proliferation and early differentiation into immature neurons were unaltered by conditional deletion of *Oxtr*. Because we observed no significant changes in cell proliferation in the DG following conditional deletion of *Oxtr* by an in vivo BrdU incorporation assay, it might be possible that the level of endogenous OXT in the hippocampus is not enough to exert a tonic stimulation of progenitor cell proliferation. Indeed, peripheral OXT administration was previously found to enhance cell proliferation in the adult rat DG in a dose-dependent manner^17^.

Newly generated DGCs exhibit features of dendrites as early as 2 weeks after birth and display overall morphological and functional properties of fully mature GCs at 6 weeks of cell age^29, 30^. In addition, the rate of newly generated DGC maturation highly correlates with the pattern of neuronal activity^31^. We demonstrated that conditional deletion of *Oxtr* decreased dendritic complexity of newly generated DGCs, as shown by decreased dendritic length and branching, at 14 dpi. However, the suppressive effect of *Oxtr* deletion on dendrite maturation of newly generated DGCs is short lived and dendritic complexity returns to normal levels at 28 dpi. In accordance with morphological observations, our electrophysiological data indicate that the maturation of excitatory synaptic transmission and the developmentally regulated shift in the actions of GABA from excitation to inhibition on newly generated DGCs were delayed by conditional deletion of *Oxtr*. These data suggest that, although endogenous OXT signaling has effects on morphological properties of newly generated DGCs during their early development, some compensatory mechanisms may exist during late developmental stages to normalize the morphological and physiological changes caused by *Oxtr* deletion.

A pressing question that follows these observations is how conditional deletion of *Oxtr* from hippocampal excitatory neurons impairs neurogenesis in the DG. Our results indicate that CA3 pyramidal neurons exert the stimulatory effect of OXT on neurogenesis in the DG of adult mice. By using optogenetic stimulation and immunofluorescence imaging, we found that CA3 pyramidal neurons functionally project to DGCs. In support of our observations, a recent viral-genetic tracing study has provided evidence that newly generated DGCs receives excitatory afferents from CA3 pyramidal neurons^32^. In addition, lesions to the CA3 region of the hippocampus have been found to decrease the survival of newly generated DGCs^21^. Thus, excitatory inputs from CA3 to newly generated DGCs are crucial in maintaining the survival of newly generated DGCs. How do CA3 pyramidal neurons influence the survival of newly generated DGCs? Considering that network activity plays a critical role in the survival of newly generated neurons in the adult brain^33–35^, it is therefore possible that OXT may increase the excitability of CA3 pyramidal neurons, thereby promoting network activity and newly generated DGC survival. In support of this view, we observed that bath application OXT induced a membrane depolarization and increased the number of action potentials in response to postsynaptic depolarizing current injection, suggesting a role for OXT in regulating the excitability of CA3 pyramidal neurons. Our findings that chemogenetic activation of CA3 pyramidal neurons enhanced the survival of newly generated DGCs in *Oxtr* ^*−/−*^ mice, whereas chemogenetic inhibition of CA3 pyramidal neurons decreased the number of newly generated DGCs in WT mice, clearly confirm that CA3 pyramidal neuron activity can influence the survival of newly generated DGCs.

OXT may exert its biological effects through the activation of OXTR-expressing interneurons. It was previously demonstrated that OXT activates a subpopulation of GABAergic interneurons within the lateral subdivision of the central nucleus of the amygdala (CeA) that inhibits activity in medial CeA neurons, thereby attenuating conditioned fear responses^36, 37^. Furthermore, OXT has been shown to enhance hippocampal spike transmission by modulating fast-spiking interneurons^16^. Very recent work also reports that OXT can activate a small subset of fast-spiking hilar GABAergic interneurons, which provide local inhibition to mossy cells in the DG^38^. In this context, our immunofluorescent staining study demonstrates that a small fraction of hilar OXTR-expressing cells are also immunoreactive for GAD67, calretinin, or parvalbumin. It is worth noting that, although parvalbumin interneuron activation has been shown to effectively promote newborn neuronal progeny survival and development via GABA signaling^34, 39^, this mechanism cannot account for the observed impaired survival of newly generated DGCs in our *Oxtr* conditional knockout mice. Indeed, we conditionally deleted *Oxtr* under the control of the CaMKIIα promotor, which was not expressed in parvalbumin-expressing interneuron^40^. A recent study has demonstrated that CaMKIIα was expressed in ~40% calretinin-immunoreactive neurons in the DG of 4- to 5-week-old mice^41^; however, we rarely detected double-labeled neurons immunoreactive for CaMKIIα and calretinin in 10-week-old mice (Lin et al., unpublished observation). This may exclude a role of OXTR-expressing calretinin interneurons in mediating the effects of conditional deletion of *Oxtr* on adult DG neurogenesis. Since *Oxtr* deletion in conditional *Oxtr* knockout mice was not restricted to the hippocampus, we could not exclude the possibility that the observed impairment of adult neurogenesis in *Oxtr* ^*−/−*^ mice may be partially mediated through OXTR-expressing excitatory neurons within extra-hippocampal regions that also provide afferent inputs to the DG, such as the amygdala^2^. Although we have conducted site-specific manipulation experiments to confirm the role of hippocampal CA3 OXT signaling in regulating DG neurogenesis, our study cannot definitively rule out the possibility that CA3 pyramidal neurons may, in part, control DG neurogenesis indirectly through the regulation of subsets of hilar GABAergic interneuron activity. Further research is warranted to test this possibility.

In conclusion, we provide compelling evidence that conditional deletion of *Oxtr* from hippocampal excitatory neurons results in reduced survival and impaired maturation of newly generated DGCs, suggesting a potential role for endogenous OXTR signaling in regulating adult hippocampal neurogenesis. Our results also reveal that CA3 pyramidal neurons play an important role in the regulation of adult hippocampal neurogenesis and OXT controls adult hippocampal neurogenesis through an indirect non-cell autonomous mechanism by OXTR expressed in CA3 pyramidal neurons. Given the prominent role of endogenous OXT signaling in enhancing adult hippocampal neurogenesis, we therefore speculate that it may serve as a potential therapeutic target for neurodegenerative disorders.

## Methods

### Animals

Adult male C57BL/6 (8–12 weeks old), homozygous *Oxtr*-floxed (*Oxtr* ^*f/f*^), and CaMKIIα-Cre transgenic mice were originally obtained from The Jackson Laboratory or and bred within our animal facility. *Oxtr* ^*f/f*^ mice were crossed to CaMKIIα-Cre mice to generate *Oxtr* conditional knockout (*Oxtr* ^*−/−*^) mice in the C57BL/6 genetic background. The heterozygous *Oxtr* ^Venus-Neo/+^ mice were generated as described previously^20^. Mice were genotyped by a PCR-based method using genomic DNA isolated from tail samples. The primers used were as follows: *Oxtr*, forward (5′-GGCTCAGGCTTTCTCTACTT-3′) and reverse (5′-GTTGGGAACAGCGGTGATTA-3′); *Cre*, forward (5′-GCGGTCTGGCAGTAAAAACTATC-3′) and reverse (5′-GTGAAACAGCATTGCTGTCAACTT-3′); *Venus*, forward (5′-CTGACCCTGAAGCTGATCT-3′) and reverse (5′-GGTAGCTCAGGTAGTGGTTG-3′). Mice were housed in groups of three in a humidity- and temperature-controlled (25 ± 1 °C) vivarium on a 12-h light/dark cycle with access to food and water ad libitum. All experimental procedures were conducted in accordance with the National Institutes of Health Guide for the Care and Use of Laboratory Animals and were approved by the Institutional Animal Care and Use Committee of National Cheng Kung University. All efforts were made to minimize the number of animals used and their suffering.

### Quantitative real-time PCR (qPCR)

Total RNA was isolated from hippocampal CA2, CA3, and hypothalamus tissue lysates using a Tri Reagent kit (Molecular Research Center) and treated with RNase-free DNase (RQ1; Promega) to remove potential contamination by genomic DNA. Total RNA (2 μg) from samples was reverse transcribed using a SuperScript cDNA synthesis kit (Invitrogen). qPCR was performed on the Roche LightCycler instrument (Roche Diagnostics) using the FastStart DNA Master SYBR Green I kit (Roche Applied Science) according to the manufacturer’s instructions. The primers used in this experiment for *Oxtr* were as follows: forward (5′-TTCTTCGTGCAGATGTGGAG-3′) and reverse (5′-CCTTCAGGTACCGAGCAGAG-3′). The PCR reactions were run for 40 cycles. Each amplification cycle included denaturation at 95 °C for 20 s, annealing at 58 °C for 20 s, and extension at 72 °C for 40 s. All reactions were repeated in duplicate and data were analyzed by lightcycler relative quantification software. The expression levels of the target gene were normalized to *β*-*actin* rRNA.

### Immunofluorescence

*Oxtr* ^Venus-Neo/+^ mice were deeply anesthetized with sodium pentobarbital (50 mg/kg, intraperitoneally) and perfused transcardially with PBS and 4% paraformaldehyde. After the perfusion, brains were removed and continue to fix in 4% paraformaldehyde for 24 h at 4 °C and then transferred to the solution containing 30% sucrose that immersed in 4 °C for at least 48 h before slicing. Coronal slices were sectioned to a 40 μm thickness, washed with 0.3 % Triton X-100, and then incubated for blocking with solution containing 3% goat serum in PBS. After blocking, the sections were incubated in the primary antibodies against nestin (1:500; Millipore, MAB353), Ki67 (1:500; Abcam, ab15580), DCX (1:500; Cell Signaling Technology, #4604), NeuN (1:1000; Millipore, ABN78), calretinin (1:500; Millipore, MAB1568), parvalbumin (1:500; Swant, PV235), calbindin (1:500; Millipore, AB1778), CaMKIIα (1:500; Novas Biologicals, NB100-81830), or GAD67 (1:500, Millipore, MAB5406). Finally, sections were washed with TBS-T (10 mM Tris-HCl, 150 mM NaCl, and 0.025% Tween 20; pH 7.4) and then incubated with the secondary Alexa Fluor 568 antibody (Life Technologies) for 2 h at room temperature in blocking buffer. The immunostained sections were collected on separate gelatin-subbed glass slides, rinsed extensively in PBS, and mounted with ProLong Gold Antifade Reagent (Invitrogen). Fluorescence images of neurons were obtained using an Olympus FluoView FV1000 confocal microscope with sequential acquisition setting at a resolution of 1024 × 1024 pixels, z-stack with 15–20 optical sections. All images were imported into NIH ImageJ software (National Institutes of Health) for analysis, and all the parameters used were kept consistent during capturing.

### BrdU injection and quantification of newly generated DGCs

For proliferation assay, mice were received single pulse of BrdU (50 mg/kg; Sigma-Aldrich) injection. After 2 h, mice were perfused with 4% paraformaldehyde in PBS (pH 7.4) and brain tissue collected. For quantification of the survival neurons, mice were injected six times intraperitoneally with BrdU at 12 h intervals and killed by transcardial perfusion after 2 and 4 weeks after the last BrdU injection. For studying the effect of CA3 neuronal activity on newborn neurons survival, AAV-hM3Dq or AAV-hM4Di was bilaterally injected into the CA3 region. One week later, mice were injected six times intraperitoneally with BrdU at 12 h interval and daily intraperitoneally injected with CNO (10 mg/kg; Sigma-Aldrich) for continuous 2 weeks until killing. The entire dentate gyrus (DG, −0.9 to −4.2 mm from bregma) was sectioned coronally at a thickness of 40 μm using a sliding microtome (Leica SM2010R).

Fluorescent immunolabelling was used for counting the number of newly generated DGCs. For BrdU/Ki67, BrdU/DCX, or BrdU/NeuN double labeling, free-floating sections were denatured in 10 mM saline-sodium citrate buffer at 85 °C for 20 min and then incubated at 37 °C for 30 min in 2 N HCl. Sections were rinsed twice for 5 min at 25 °C in 0.1 M Na borate (pH 8.5) and then incubated in the primary antibodies against BrdU (1:500; Millipore, MAB4072), Ki67(1:500; Abcam, ab15580), DCX (1:500; Cell Signaling Technology, #4604), or NeuN (1:1000; Millipore, ABN78) overnight at 4 °C in PBS with 0.1% Triton X-100. Finally, sections were washed with PBS and then incubated with the secondary Alexa Fluor 488, 568, or 647 antibodies (Life Technologies) for 2 h at room temperature. The nuclei were visualized using 4′,6-diaminodino-2-phenylindole (DAPI, 1:5000; Sigma-Aldrich) staining. The immunostained sections were collected on separate gelatin-subbed glass slides, rinsed extensively in PBS, and mounted with ProLong Gold Antifade Reagent (Invitrogen).

Quantification of BrdU-labeled cells was performed using a modified unbiased stereology protocol as described previously^42, 43^. Every sixth section covering the entire DG was processed for BrdU immunohistochemistry. All BrdU-labeled cells in the granule cell layer, subgranular zone, and hilus were counted under fluorescent illumination at ×400 using an Olympus BX51 microscope coupled to an Olympus DP70 digital camera. Unbiased stereologic analysis was applied to count BrdU-labeled cells^43^. Total cell numbers were estimated by multiplying the number of cells counted in every sixth section by six.

### Fluorescent in situ hybridization

Fluorescent in situ hybridization was performed using RNAscope^®^ Multiplex Fluorescent Reagent Kit 2.0 according to the manufacturer’s instructions (Advanced Cell Diagnostics). Briefly, brain sections (16 μm) were fixed in 4% paraformaldehyde for 15 min and dehydrated through graded ethanol solutions (50, 70, and 100%) for 5 min each. Sections were subjected to reagent pretreat 3 at 25 °C for 30 min and then hybridized with probes at 40 °C for 2 h in a humidified oven. The *Oxtr*-O1 probe (Cat# 454011) and the *CaMKIIα* probe (Cat# 445231) were used to target exon 3 of the *Oxtr* mRNA and *CaMKIIα* mRNA. After hybridization, brain sections were sequentially applied with a series of probe signal amplification steps, rinsed with ACD wash buffer twice for 2 min between each step and finally counterstained with DAPI and mounted with VECTASHIELD antifade mounting medium (Vector Laboratories) containing DAPI (1:5000; Sigma-Aldrich).

### Retrovirus production, stereotaxic injection, and analysis

Engineered self-inactivating murine retroviruses expressing EGFP were used to label proliferating cells and their progeny in the DG of adult mice as described previously^43, 45^. High titers of engineered retroviruses (1 × 10^9^ unit/ml) were produced by co-transfection of retroviral vectors and VSV-G into HEK293GP cells followed by ultracentrifugation of viral supernatant. The Cre recombinase (Addgene, plasmid #20781) was cloned into a retroviral expression vector Ubi-X-2A-EGFP.

Adult male WT or homozygous *Oxtr* ^*−/−*^ mice were anesthetized and the purified retroviruses were stereotaxically injected into the DG at 4 sites (0.5 μl/site at 0.25 μl/min) with the following coordinates (anterior-posterior = −2 mm from bregma, lateral ±1.6 mm, ventral = 2.5 mm; anterior-posterior 3 mm from bregma, lateral = ±2.6 mm, ventral = 3.2 mm) in accordance with the description by Franklin and Paxinos^46^. Mice were killed at 14 and 28 dpi for morphological analysis. Coronal brain sections (40 μm thick) were prepared from viral-injected mice and processed for immunostaining as previously described^42, 44^. Sections were incubated for 30 min in DAPI before washing and mounting. Images were acquired on an Olympus FluoView FV1200MPE multiphoton confocal system using a multi-track configuration. Z-series stack of confocal images were taken and a single confocal image slice with the largest soma area for individual EGFP^+^ neurons was chosen for quantification using NIH ImageJ program.

For dendritic development analysis, three-dimensional reconstruction of the entire processes of each neuron was made from Z-series stacks of confocal images. The projection images were semi-automatically traced with NIH ImageJ (http://rsb.info.nih.gov/ij/) using the NeuronJ Plugin. All EGFP^+^ DGCs with intact dendritic trees were analyzed for total dendritic length and branch number. Dendritic complexity was quantified by Sholl analysis by counting the number of dendrites that crossed a series of concentric circles at 10 μm intervals from the cell soma using the Sholl analysis plug-in.

### Adeno-associated virus production

DNA plasmids encoding pAAV-CaMKIIα-hM3Dq-internal ribosomal entry site (IRES)-mCitrine (Addgene, plasmid #50466), pAAV-CaMKIIα-hM4Di-mCherry (Addgene, plasmid #50477), pAAV-CaMKIIα-hChR2(H134R)-EYFP (Addgene, plasmid #26969), pAAV-CaMKIIα-EGFP (Addgene, plasmid #50469), and pAAV-Ubi-eGFP (Addgene, plasmid #62518) were obtained from Addgene. pAAV-CaMKIIα-Cre-GFP was constructed from Cre-GFP empty vector (Addgene, plasmid #20781) into pAAV-CaMKIIα-hChR2-EYFP (Addgene, plasmid #26969). Plasmid DNA was amplified, purified, and collected using a standard plasmid maxiprep kit (Qiagen). The purified plasmids were mixed into CaCl_2_ solution with the DNA plasmid coding AAV-DJ and co-transfected into HEK293T cells using calcium phosphate precipitation methods. Transfected cells were harvested at 72 h after transfection and the virus was purified using the AAV purification mega kit (Cell Biolabs, Inc.). Viral titers were 5 × 10^12^ particles/ml and stored in aliquots at −80 °C until use.

### Retrograde tracing

For retrograde tracing of projecting neurons, fluorescence Alexa Fluor 555-conjugated CTB (0.1 μl; Thermo Fisher, Cat#C-34776) was injected bilaterally into the CA3 region rostral −2.5 mm, lateral ±2.8 mm, and ventral −2.5 mm at 0.1 μl/min by using 1 μl Hamilton syringe. The syringe was slowly retracted after additional 5 min solution diffusion. For double-labeling neuroendocrine neurons, mice were simultaneously received an intraperitoneally injection of fluoro-gold (4% w/v in 100 μl saline; Santa Cruz, Cat#SC-358883). Ten days later, the animals were perfused transcardially with PBS followed by 4% paraformaldehyde solution. Brains were then removed and cut into 40 μm-thick coronal sections with a Vibrotome and immunofluorescence stained with the primary antibody against OXT (1:500; Millipore, AB911) and fluoro-gold (1:1000; Millipore, AB153-I).

### TUNEL assay

Apoptosis examination was performed using in situ cell death detection kit (Roche, Cat#1684795) following the manufacturer’s instruction. Briefly, PFA-perfused brain sections were microwave treated in 0.1 M citrate buffer for 1 min and washed twice in PBS. For labeling, sections were incubated in TUNEL enzyme solution for 1 h at 37 °C in the dark and counterstained with DAPI. Positive controls were obtained by incubating hippocampal slices with DNase (3000 U/ml in 50 mM Tris-HCl, pH 7.5, 1 mg/ml BSA) for 15 min at 25 °C to induce DNA-strand breaks, prior to labeling procedure.

### Electrophysiological recordings

Hippocampal slices were prepared using standard procedures as described previously^43, 45^. In brief, were anesthetized with isoflurane and decapitated, and brains were rapidly removed and placed in ice-cold oxygenated sucrose cutting solution (containing (in mM): sucrose 234, KCl 2.5, CaCl_2_ 0.5, MgCl_2_ 7, NaHCO_3_ 25, NaH_2_PO_4_ 1.25, and glucose 11 at pH 7.3–7.4 and equilibrated with 95% O_2_–5% CO_2_). Hippocampal slices (250 μm) were prepared using a vibrating microtome (VT1200S; Leica) and transferred to a holding chamber of artificial cerebrospinal fluid (ACSF) (containing (in mM): NaCl 117, KCl 4.7, CaCl_2_ 2.5, MgCl_2_ 1.2, NaHCO_3_ 25, NaH_2_PO_4_ 1.2, and glucose 11 at pH 7.3–7.4 and equilibrated with 95% O_2_-5% CO_2_) and maintained at room temperature for at least 1 h before use.

For recording, slices were transferred to a submersion-type recording chamber and fixed at the glass bottom of the chamber with a nylon grid on a platinum frame. The chamber was constantly perfused with ACSF at 32.0 ± 0.5 °C with a rate of 2–3 ml/min. Conventional whole-cell and gramicidin-perforated patch-clamp recordings were made from newly generated (EGFP^+^) DGCs, mature (EGFP^−^) DGCs or CA3 pyramidal neurons by using a patch-clamp amplifier (Axopatch 200B, Molecular Devices) under infrared differential interference contrast microscope. Mature DGCs were recorded from the outer portion of the granule cell layer. Data acquisition and analysis were performed using a digitizer (Digidata 1440 A, Molecular Devices) and pCLAMP 9 software (Molecular Devices). Synaptic responses were evoked using a bipolar stainless steel stimulating electrode placed in the middle molecular layer ~100 μm away from the recorded cell. For measurement of E_GABA_ in newly generated DGCs, pharmacologically isolated GABA_A_receptor-mediated IPSCs were recorded using gramicidin-perforated patch under voltage-clamp at different holding potentials in the presence of CNQX (20 μM; Tocris Bioscience) and APV (50 μM; Tocris Bioscience) to eliminate glutamate currents. A stock solution of gramicidin (10 mg/ml) was prepared in dimethyl sulfoxide and was then diluted in the pipette solution (containing (in mM): CsCl 135, MgCl_2_ 2, EGTA 0.5, HEPES 10, pH  7.3, pH 7.3, 290–295 mOsm) to a final concentration of 25 µg/ml. After gigaseal formation, the progress of perforation was continuously monitored until the series resistance had stabilized to ≤30 MΩ. The intracellular chloride concentration was calculated according to the Nernst equation: [Cl^−^]_i_ = [Cl^−^]_o_e(E_GABA_F/RT), where the extracellular Cl^−^ concentration [Cl^−^]_o_ is 125.4 mM.

sEPSCs and mEPSCs were recorded from newly generated or mature DGCs held under voltage-clamp at −70 mV in the presence of gabazine (10 μM; Tocris Bioscience) and analyzed off-line using commercially available software (Mini Analysis 4.3; Synaptosoft). Recordings of mEPSCs were conducted in the presence of TTX (0.5 μM; Tocris Bioscience). The composition of intracellular solution was (mM): K-gluconate 120, KCl 15, HEPES 10, MgCl_2_ 4, EGTA 0.1, Na_2_ATP 4, Na_3_GTP 0.3, phosphocreatine 7, and 0.5% w/v biocytin, 280–290 mOsm without biocytin, pH 7.3 with KOH. The detection threshold for sEPSCs and mEPSCs was set at 3 pA.

To examine the effects of OXT on intrinsic membrane properties of OXTR-expressing CA3 pyramidal neurons, hippocampal slices were prepared from OXTR-Venus knock-in mice. After a stable recording of membrane potential under current-clamp condition, OXT (1 μM) was applied to the recorded cell by a 5-min bath application. In some experiments, hyperpolarizing (−50 pA) and depolarizing current pulses (100 pA) of 500 ms were injected through the recording pipette in current-clamp mode to measure neuronal excitability. The number of action potentials triggered was counted. Biocytin was routinely included in the intracellular solution to allow post hoc staining of the recorded neurons. OXT and L-371257 were purchased from Tocris Bioscience.

To confirm the expression of engineered hM3Dq or hM4Di receptors in CA3 neurons, a depolarizing current pulse (150 pA, 500 ms) was injected into the control, hM3Dq^+^ or hM4Di^+^ (hM3Dq or hM4Di expression) neurons to induce spiking. Following 10 min continuous recordings, CNO (1 μM; Sigma-Aldrich) was applied into the ACSF and a second depolarizing current (+150 pA, 500 ms) or hyperpolarizing current pulse (−50 pA, 500 ms) was injected into the hM3Dq^−^, hM3Dq^+^, hM4Di^−^, and hM4Di^+^ neurons to induce spiking in order to compare spiking before and after CNO application.

For in vitro optical stimulation, a multimode optical fiber with core diameter of 200 μm (Thorlabs), coupled to a diode-pumped solid-state laser of specific wavelength (473 nm blue laser; Laserglow Technologies), was used. The ending power on brain slices was ~5 mW/mm^2^ and synaptic responses were recorded at −70 mV in voltage-clamp mode.

### Statistical analysis

No statistical tests were used to predetermine sample sizes, but our sample sizes were consistent with the previous publication using similar approaches^22, 43^. Mice were randomly assigned to viral injection experiments, and investigators were blinded to the group allocation while performing cell number counting, morphological analysis, and electrophysiological recordings. The results are presented as mean ± s.e.m. All statistical analyses were performed using the Prism 6 software package (GraphPad software). The significance of any difference between two groups was calculated using the paired or unpaired two-tailed Student’s *t* test. One-way ANOVA or repeated measure two-way ANOVA tests were used for multiple groups’ comparison and Bonferroni’s post hoc analyses were used to assess the significance between isolated groups. Kolmogorov-Smirnov test were used in comparison of cumulative frequency distribution. *N* represents the number of animals used. Values of *P* < 0.05 were considered significant.

### Data availability

The authors declare that all data supporting the findings of this study are available within the article and its Supplementary Information File.

## Electronic supplementary material

Supplementary Information
