# Supplementary material for: Oxytocin stimulates hippocampal neurogenesis via oxytocin receptor expressed in CA3 pyramidal neurons
Source: Nat Commun. 2017 Sep 14;8:537. doi: 10.1038/s41467-017-00675-5 (PMC5599651; doi:10.1038/s41467-017-00675-5)
Supplement: Supplementary file 1 — Supplementary Information [file 41467_2017_675_MOESM1_ESM.pdf]

Description of Supplementary Files

File Name: Supplementary Information
Description: Supplementary Figures.

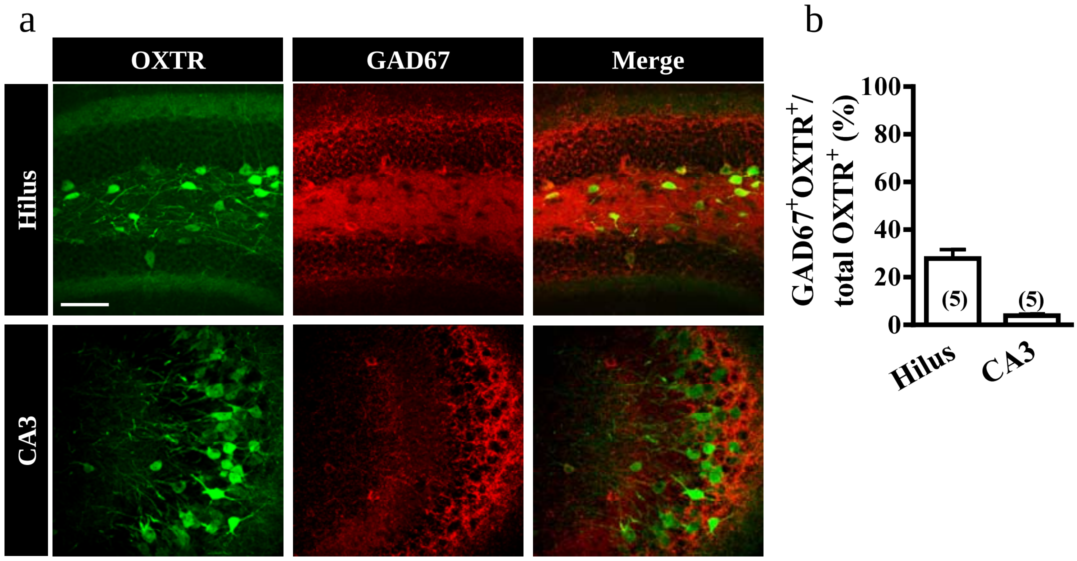

Supplementary Figure 1 | OXTR is expressed in some subsets of GAD67-expressing cells in the DG and CA3 of the hippocampus. (**a**) Double-labeled confocal immunofluorescence images showing the colocalization of OXTR (green) expression with GAD67 (red) in the hilus of the DG (upper panel) and the CA3 (lower panel) of 10-week-old *Oxtr*$^{Venus\text{-}Neo/+}$ mice. (**b**) Quantification of the percentage of GAD67^{+}OXTR^{+} among total OXTR^{+} cells in the hilus of the DG and CA3 of *Oxtr*$^{Venus\text{-}Neo/+}$ mice. The total number of mice examined is indicated by *n* in parenthesis (n = 5). Data represent the mean ± s.e.m. Scale bar, 50 μm.

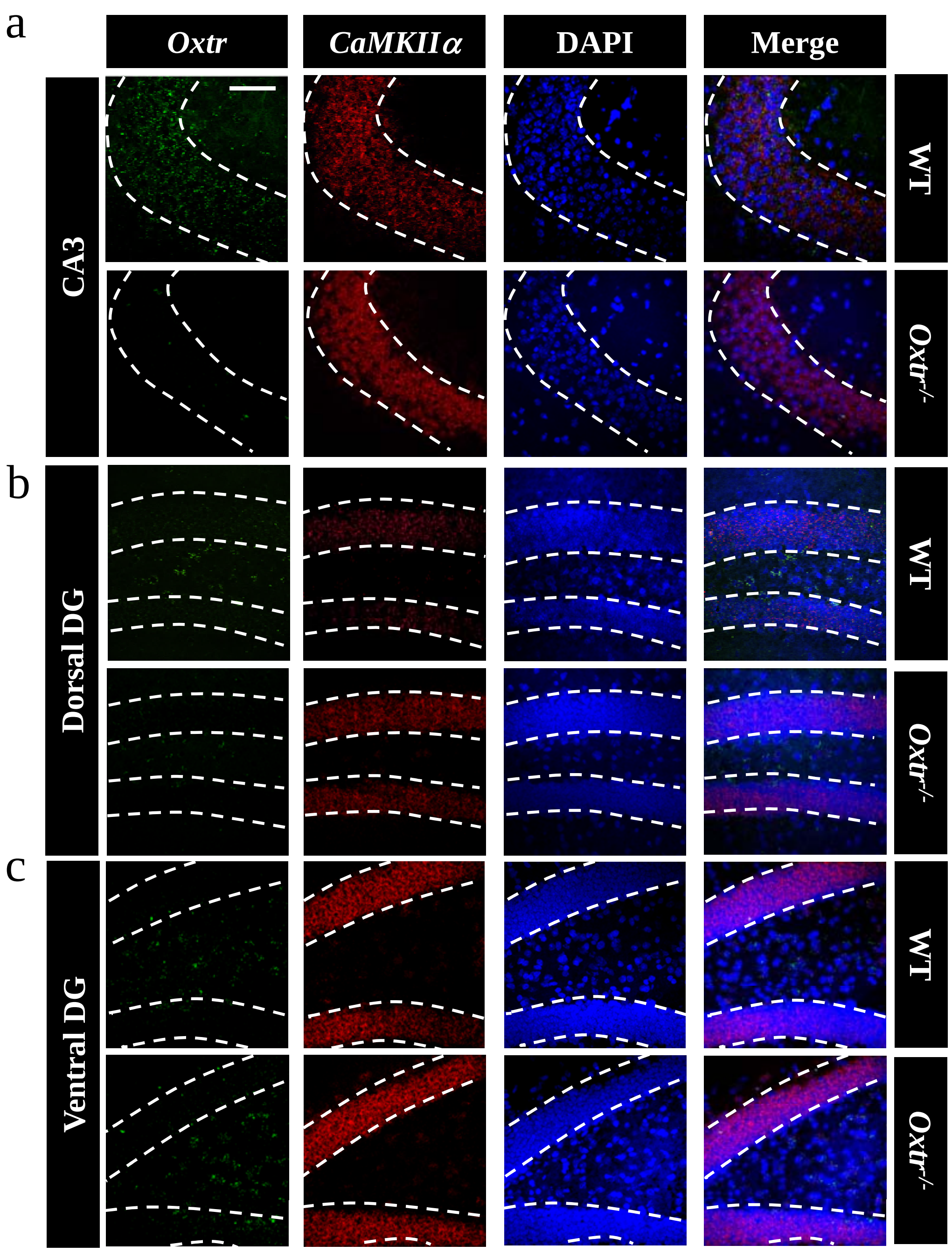

Supplementary Figure 2│ *Oxtr* is expressed in *CaMKIIα*-expressing cells in the CA3 of the hippocampus. (**a**) Dual-probe FISH showing the expression of *Oxtr* mRNA (green) and *CaMKIIα* mRNA (red) in the CA3 of WT (upper panel) and *Oxtr*$^{-/-}$ mice (lower panel) (counterstained with DAPI, blue). (**b**,**c**) Dual-probe FISH showing the expression of *Oxtr* mRNA and *CaMKIIα* mRNA in the dorsal (**b**) and ventral DG (**c**) of WT (upper panel) and *Oxtr*$^{-/-}$ mice (lower panel). Scale bar, 50 μm. Data was replicated in 4 mice.

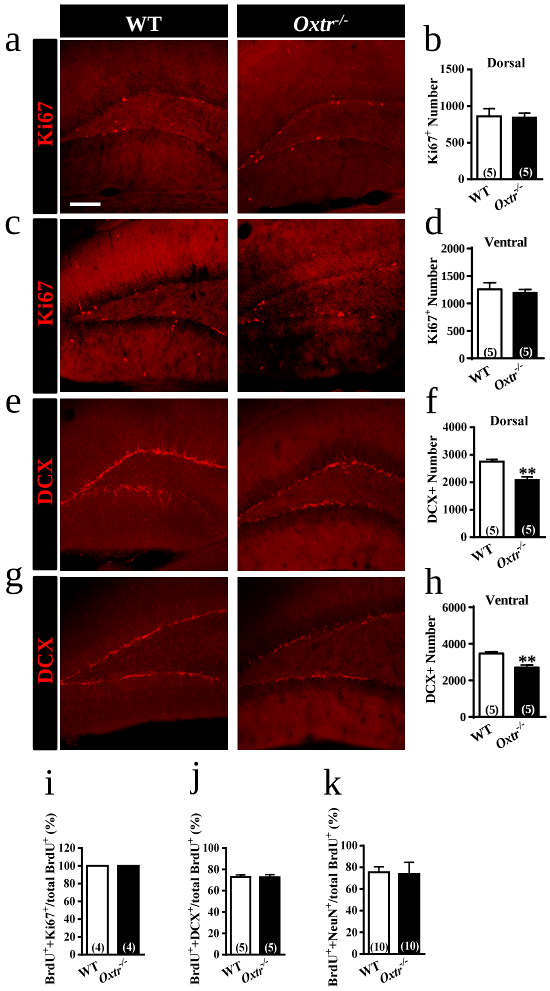

Supplementary Figure 3 | Conditional deletion of *Oxtr* impairs the survival of newly generated DGCs. (**a**,**c**) Representative immunofluorescence images of dorsal (**a**) or ventral hippocampal sections (**c**) from WT and *Oxtr*$^{-/-}$ mice stained for Ki67 (red) at 2 hours after BrdU injection. (**b**,**d**) Quantification of the total number of Ki67^{+} cells in dorsal (**b**) or ventral DG (**d**) of WT and *Oxtr*$^{-/-}$ mice at 2 hours after BrdU injection (n = 5 mice per genotype, unpaired two-tailed Student's t-test). (**e**,**g**) Representative immunofluorescence images of dorsal (**e**) or ventral hippocampal sections (**g**) from WT and *Oxtr*$^{-/-}$ mice stained for DCX (red) at 14 days after the last BrdU injection. (**f**,**h**) Quantification of the total number of DCX^{+} cells in dorsal (**f**) or ventral DG (**h**) of WT and *Oxtr*$^{-/-}$ mice at 14 days after the last BrdU injection (n = 5 mice per genotype; *P < 0.05, unpaired two-tailed Student's t-test). (**i**) Quantification of the percentage of BrdU^{+}Ki67^{+} among total BrdU^{+} cells in the dentate gyrus of WT and *Oxtr*$^{-/-}$ mice at 2 hours after BrdU injection (n = 4 mice per genotype, unpaired two-tailed Student's t-test). (**j**) Quantification of the percentage of BrdU^{+}DCX^{+} among total BrdU^{+} cells in the DG of WT and *Oxtr*$^{-/-}$ mice at 14 day after the last BrdU injection (n = 5 mice per genotype, unpaired two-tailed Student's t-test). (**k**) Quantification of the percentage of BrdU^{+}NeuN^{+} among total BrdU^{+} cells in the DG of WT and *Oxtr*$^{-/-}$ mice at 28 day after the last BrdU injection (n = 10 mice per genotype, unpaired two-tailed Student's t-test). Scale bar, 100 μm. Data represent the mean ± s.e.m.

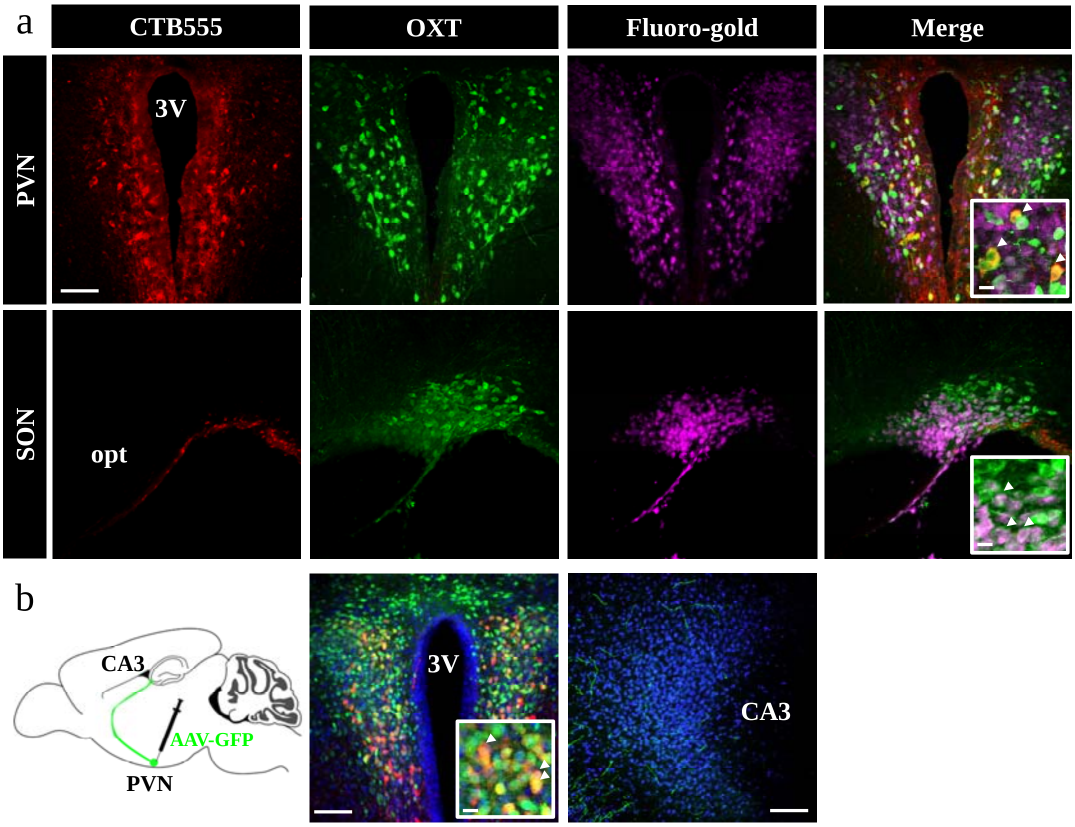

Supplementary Figure 4 | PVN cells project to hippocampal CA3. (**a**) CTB was injected into the CA3. The neurosecretory neurons were labeled with intraperitoneal injection of the fluoro-gold (4% w/v in 100 μl saline). Immunoreactivity for CTB (red) was clearly detected in fluoro-gold$^-$ (pink) and OXT$^+$ neurons (green) of the PVN (upper panel), but not the SON (bottom panel). The insets represent high-magnification images. Scale bar, 100 μm and 20 μm (rectangle amplification). (**b**) Left panel, a schematic of the approach to observe OXT fibers in the CA3 region by injection of AAV-Ubi-GFP into PVN. Middle panel, OXT neurons in the PVN labeled with anti-OXT antibody (red) were infected with AAV-Ubi-GFP and couterstained with and DAPI (blue). Right panel, a representative image showing the distribution of GFP-labeling of fibers in the CA3. Sections were couterstained with DAPI (blue). Scale bar, 100 μm and 20 μm (rectangle amplification). 3V, third ventricle; opt, optic tract. Data was replicated in 4 mice.

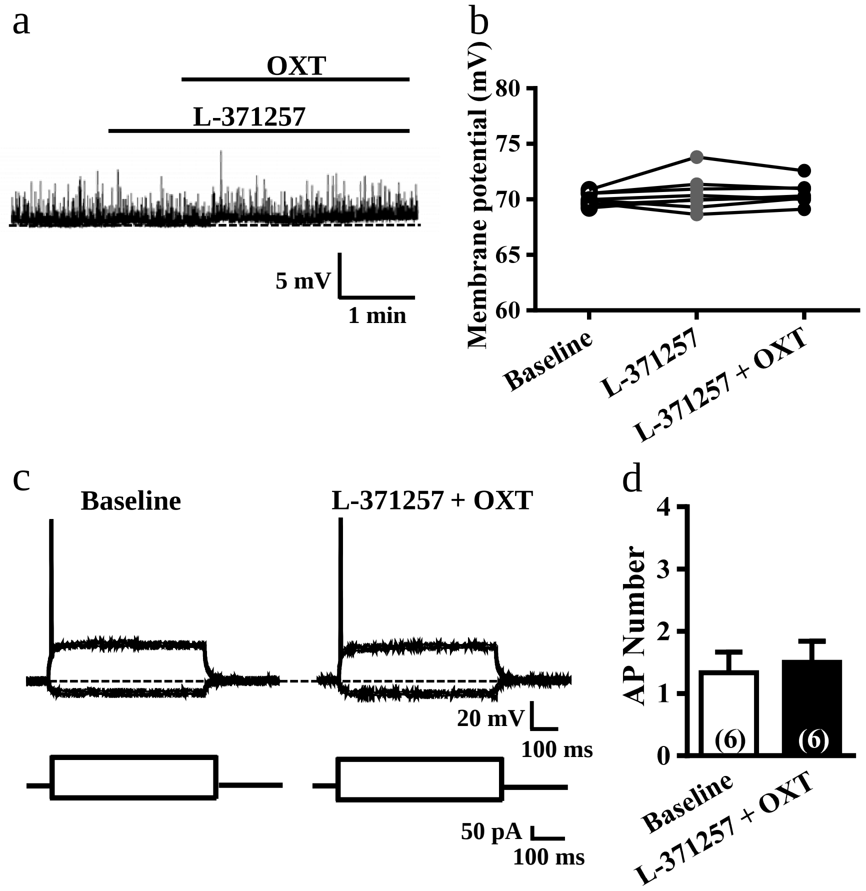

Supplementary Figure 5│ OXTR antagonist prevents the enhancing effects on OXT on CA3 pyramidal neuron excitability. (**a,b**) Representative traces and summary data showing that pretreatment of the hippocampal slices with L-371257 (1 μM) prevented OXT (1 μM)-induced membrane depolarization in hippocampal CA3 pyramidal neurons from WT mice (*n* = 8 neurons from 4 mice, One-way ANOVA). (**c,d**) Representative traces and summary data showing that pretreatment of the hippocampal slices with L-371257 (1 μM) prevented the effect of OXT (1 μM) on action potential firing responses elicited by depolarizing current injection in hippocampal CA3 pyramidal neurons from WT mice (*n* = 6 neurons from 4 mice, paired two-tailed Student's *t*-test). Data represent the mean ± s.e.m.

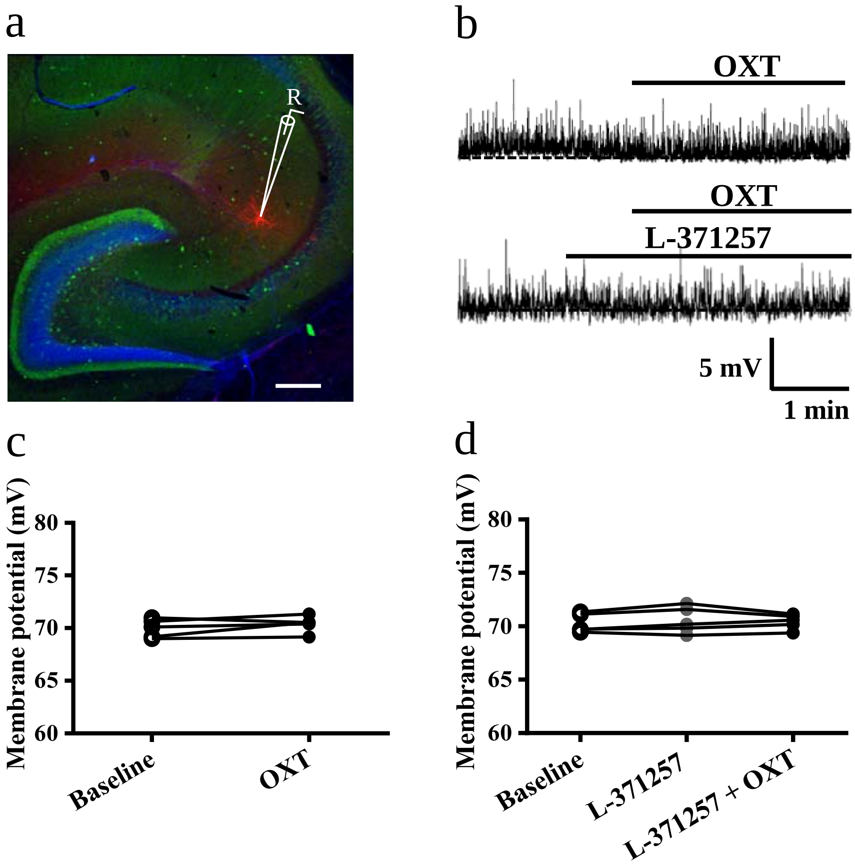

Supplementary Figure 6│ OXT does not affect the intrinsic excitability of CA3 interneurons. (**a**) OXTR$^-$ interneurons (red) were targeted for recording, filled with biocytin, and reacted with avidin-rhodamine to allow *post hoc* reconstruction in slice from *Oxtr*$^{\text{Venus-Neo/+}}$ mice. (counterstained with DAPI, blue). Scale bar, 150 µm. (**b**-**d**) Representative traces and summary data showing the effects of OXT (1 µM) on membrane potential of OXTR$^-$ interneurons in the absence (n = 5 neurons from 3 mice, paired two-tailed Student's t-test) (**c**) or presence of L-371257 (1 µM, n = 5 neurons from 3 mice, One-way ANOVA) (**d**). Data represent the mean ± s.e.m.

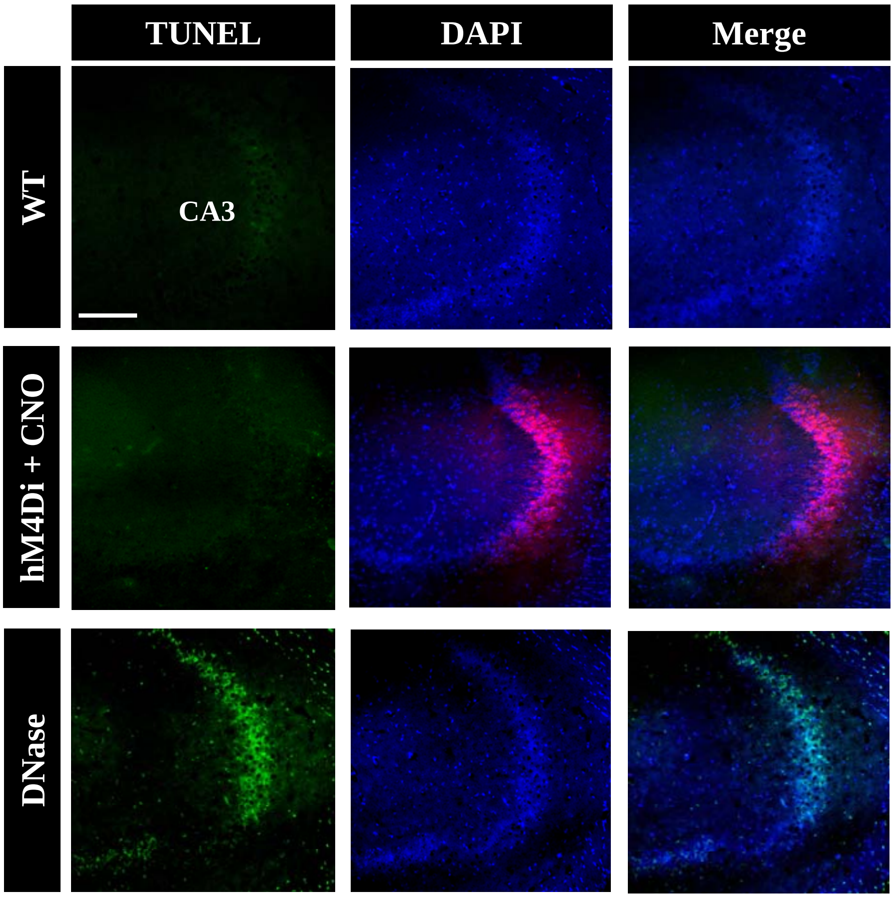

Supplementary Figure 7│ TUNEL staining in the CA3 of chronic CNO-treated mice. Representative micrographs showing the expression of TUNEL-positive cells (green) in the CA3 of WT mice without (upper panel) or with hM3Dq + CNO treatment (middel panel). Two weeks after stereotaxic injection of AAV-hM4Di (red) into the CA3 region, mice were administered daily intraperitoneal injection of CNO (10 mg/kg) for 2 weeks. Positive controls were obtained by incubating hippocampal slices with DNase (3000 U/ml in 50 mM Tris-HCl, pH 7.5, 1 mg/ml BSA) for 15 min at 25 ºC to induce DNA strand breaks, prior to labeling procedure. Nuclei were counterstained with DAPI (blue). Scale bar, 150 μm. Data was replicated in 4 mice.

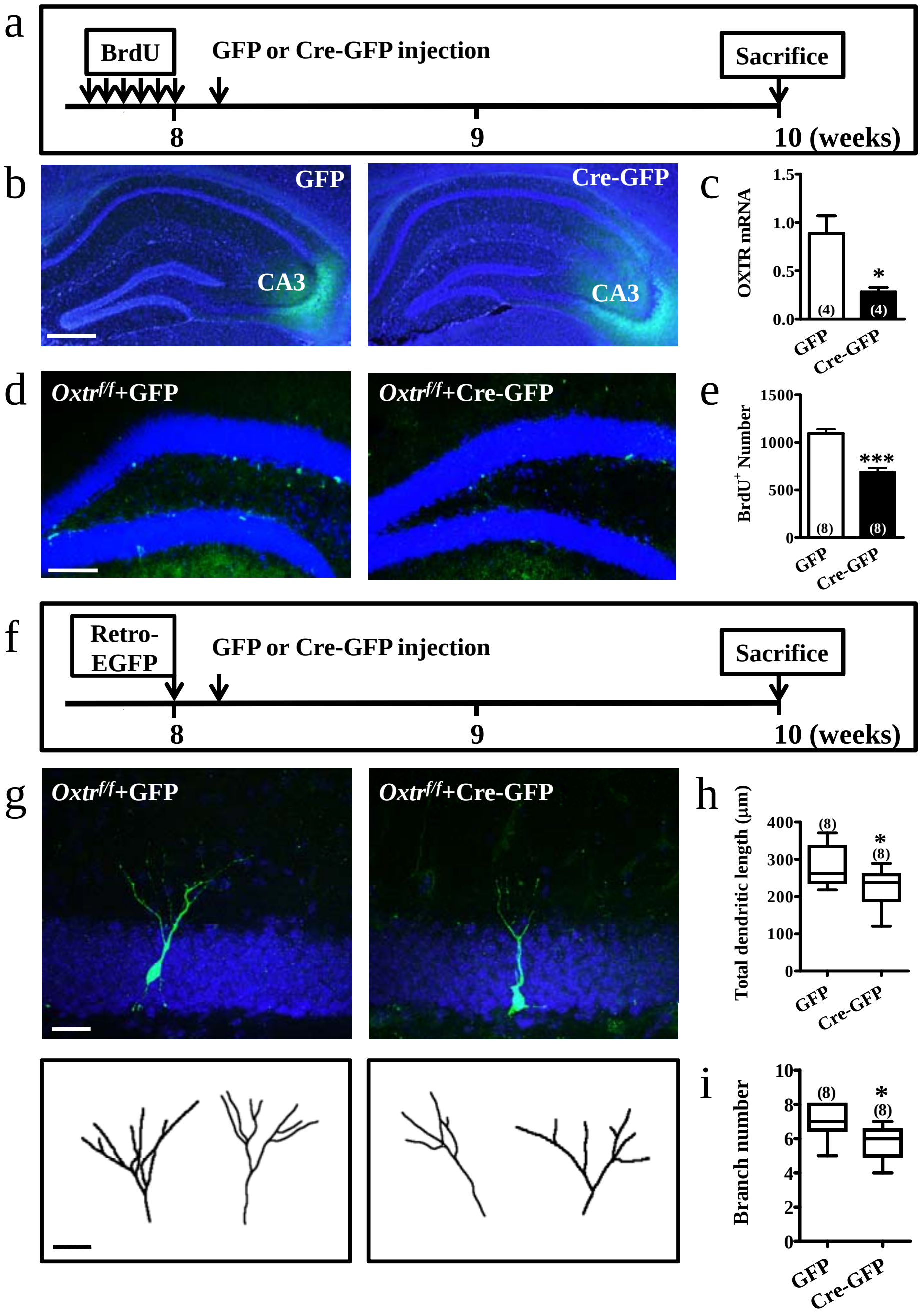

Supplementary Figure 8 | Conditional deletion of CA3 *Oxtr* impairs the survival and morphological maturation of newly generated DGCs. (**a**) Schematic representation of the experimental designs for comparing the survival and morphological maturation of newly generated DGCs in *Oxtr*$^{f/f}$ mice treated with AAV-GFP or AAV-Cre-GFP injection in the CA3 region. One day after 6 times intaperitoneally injection of BrdU (50 mg/kg) at 12 h intervals or retro-EGFP microinjection into the DG hilus, mice were stereotaxic injected AAV-GFP or AAV-Cre-GFP into the CA3 region. The number of BrdU^{+} cells were counted two weeks after the last BrdU injection. (**b**) Representative micrographs showing the expression of GFP (left) and Cre-GFP (right) in the CA3. Scale bar, 500 μm. **(c)** Quantitative real-time PCR of *Oxtr* mRNA in the CA3 region from *Oxtr*$^{f/f}$ + GFP and *Oxtr*$^{f/f}$ + Cre-GFP mice (n = 4 mice for each treatment; *p < 0.05, unpaired two-tailed

Student's *t*-test). (d) Representative immunofluorescence images of hippocampal sections from *Oxtr*$^{f/f}$ + GFP and *Oxtr*$^{f/f}$ + Cre-GFP mice stained for BrdU (green) 14 days after BrdU injection. Scale bar, 100 µm. **(e)** Quantification of the total number of BrdU^{+} cells in the DG of *Oxtr*$^{f/f}$ + GFP and *Oxtr*$^{f/f}$ + Cre-GFP mice at two weeks after BrdU injection (*n* = 8 mice for each treatment; ***P < 0.001, unpaired two-tailed Student's *t*-test). **(f)** Schematic representation of the experimental designs for comparing the morphological maturation of newly generated DGCs in *Oxtr*$^{f/f}$ mice treated with AAV-GFP or AAV-Cre-GFP injection in the CA3 region by using retrovirus-mediated labeling strategy. Engineered retroviruses expressing EGFP were stereotaxically microinjected into the hilus of the DG one day before AAV-GFP or AAV-Cre-GFP treatment. The dendritic arborization of EGFP^{+} DGCs was measured at 14 dpi. **(g)** Representative images showing Retro-EGFP^{+} DGCs in *Oxtr*$^{f/f}$ + AAV-GFP and *Oxtr*$^{f/f}$ + AAV-Cre-GFP mice at 14 dpi. Scale bar, 30 µm. (**h**,**i**) Summary bar graphs depicting (**h**) the total dendritic length and (**i**) branch number of EGFP^{+} DGCs from *Oxtr*$^{f/f}$ + GFP and *Oxtr*$^{f/f}$ + Cre-GFP mice at 14 dpi (*n* = 8 neurons from 4 mice for each treatment; *P < 0.05, unpaired two-tailed Student's *t*-test). Data represent the mean ± s.e.m.
